# Exome sequencing improves genetic diagnosis of structural fetal abnormalities revealed by ultrasound

**DOI:** 10.1093/hmg/ddu038

**Published:** 2014-01-29

**Authors:** Keren J. Carss, Sarah C. Hillman, Vijaya Parthiban, Dominic J. McMullan, Eamonn R. Maher, Mark D. Kilby, Matthew E. Hurles

**Affiliations:** 1 Wellcome Trust Sanger Institute, Wellcome Trust Genome Campus, Hinxton, Cambridgeshire CB10 1SA, UK; 2 School of Clinical and Experimental Medicine (Birmingham Centre for Women's and Children's Health), College of Medical and Dental Sciences, University of Birmingham, Edgbaston, Birmingham B15 2TT, UK; 3 West Midlands Regional Genetics Laboratory, Birmingham Women's NHS Trust, Edgbaston, Birmingham B15 2TG, UK; 4 Fetal Medicine Centre, Birmingham Women's Foundation Trust, Edgbaston, Birmingham B15 2TG, UK

## Abstract

The genetic etiology of non-aneuploid fetal structural abnormalities is typically investigated by karyotyping and array-based detection of microscopically detectable rearrangements, and submicroscopic copy-number variants (CNVs), which collectively yield a pathogenic finding in up to 10% of cases. We propose that exome sequencing may substantially increase the identification of underlying etiologies. We performed exome sequencing on a cohort of 30 non-aneuploid fetuses and neonates (along with their parents) with diverse structural abnormalities first identified by prenatal ultrasound. We identified candidate pathogenic variants with a range of inheritance models, and evaluated these in the context of detailed phenotypic information. We identified 35 *de novo* single-nucleotide variants (SNVs), small indels, deletions or duplications, of which three (accounting for 10% of the cohort) are highly likely to be causative. These are *de novo* missense variants in *FGFR3* and *COL2A1*, and a *de novo* 16.8 kb deletion that includes most of *OFD1.* In five further cases (17%) we identified *de novo* or inherited recessive or X-linked variants in plausible candidate genes, which require additional validation to determine pathogenicity. Our diagnostic yield of 10% is comparable to, and supplementary to, the diagnostic yield of existing microarray testing for large chromosomal rearrangements and targeted CNV detection. The *de novo* nature of these events could enable couples to be counseled as to their low recurrence risk. This study outlines the way for a substantial improvement in the diagnostic yield of prenatal genetic abnormalities through the application of next-generation sequencing.

## INTRODUCTION

Fetal abnormalities detected using ultrasound examination range from minor findings (i.e. dysmorphic facial features) to major, potentially lethal, multisystem abnormalities. The underlying etiology is diverse and includes environmental and genetic factors. Congenital structural anomalies affect 2.2% of births in UK and Wales (1), and the prevalence of structural fetal anomalies is likely to be even higher than this, because a proportion of these pregnancies are associated with fetal loss.

Typically, the genetic etiology of fetal abnormalities is investigated (prenatally or postnatally) by a combination of karyotyping, fluorescence *in situ* hybridization (FISH) and array-based comparative genomic hybridization (aCGH). Conventional full karyotyping may detect numerical as well as unbalanced and balanced structural rearrangements >5–10 Mb. Submicroscopic chromosomal anomalies may be diagnosed using FISH, but this is highly limited in being a ‘targeted’ approach, requiring knowledge that a specific karyotypic rearrangement is associated with a specific malformation. The popularity of aCGH in prenatal diagnostics is increasing. The sensitivity for detection is dependent upon the platform chosen and the indication for testing. A recent systematic review reported a prenatal detection rate of 10% (95% confidence interval (CI), 8–13) above conventional karyotyping in fetuses with structural malformations (2). This is consistent with studies estimating the diagnostic rate of aCGH in fetuses with structural abnormalities as 6–10% (3,4). However, aCGH is limited to the detection of copy-number variants (CNVs) of >10–100 kb in specifically targeted regions.

Next-generation sequencing (NGS) is becoming an invaluable tool not just for disease gene discovery in a research setting, but also for clinical diagnostics. The diagnostic yield of exome sequencing for patients with Mendelian diseases is 25% (5), suggesting that it might complement conventional prenatal diagnostic techniques. NGS could improve prenatal diagnostic yield by identifying pathogenic genetic variants that are below the resolution of aCGH platforms in current clinical use, and by localizing breakpoints of cytogenetically balanced chromosomal rearrangements to individual genes (6). To date, two studies have described the clinical use of NGS to make a diagnosis for a single fetus (5,6), and one study of a larger cohort reported low-coverage NGS for identifying prenatal aneuploidy and unbalanced chromosomal rearrangements (7). In our proof-of-principle study of 30 fetuses and neonates with structural anomalies first identified by ultrasound, we illustrate the power of whole-exome sequencing for identifying variants (single-nucleotide variants (SNVs), indels and CNVs) that potentially cause abnormal fetal development.

## RESULTS

### Exome sequencing

Exome sequencing in 30 fetuses and neonates with a diverse range of structural abnormalities diagnosed at prenatal ultrasound, along with their parents, was performed (a total of 86 individuals). The mean depth of coverage of the targeted coding regions was 103×. A mean of 92.7% of bases in the targeted coding regions were covered by at least 10 reads (Supplementary Material, Table S1 and Figs S1–S3). No parental phenotypic abnormalities were reported that might be related to the fetal abnormalities, suggesting dominant inheritance is unlikely. We therefore evaluated different classes of potentially pathogenic, rare coding variants under dominant *de novo*, recessive and X-linked modes of inheritance, through systematic manual curation of the existing literature, to classify variants into one of three categories: highly likely to be causal, possibly causal or unknown. For the three non-sporadic cases (the siblings F27 and F33, and F2, who has a similarly affected sibling not included in this study), all of which are female, we consider a recessive mode of inheritance most likely. We nevertheless investigated all the mutation classes described above.

### *De novo* SNVs and indels

We identified potential *de novo* SNVs and indels with high sensitivity, and inevitably low specificity, yielding a list of 77 candidate *de novo* coding or splicing variants (mean = 2.6 per fetus, range = 0–5). We attempted to validate all of these by capillary sequencing of whole genome amplified genomic DNA, irrespective of their predicted functional consequence. We validated 34 as being truly *de novo* (Table 1 and Supplementary Material, Table S2). This is a mean of 1.13 per fetal exome (range 0–4), which is within the expected range from the known germline mutation rate and NGS of other disease cohorts (8–11). These mutations include identical *PPFIBP2* mutations in the monozygotic twins F3 and F16, with the result that there are 33 independent *de novo* variants.

**Table 1.** Candidate genes identified in 27 fetuses with structural abnormalities

ID	Gender	Genes with *de novo* mutations	Genes with inherited autosomal mutations (recessive, compound heterozygous)	Genes with inherited autosomal mutations (recessive, homozygous)	Genes with inherited mutations on X chromosome (hemizygous)	Genes in CNVs
F1	Male	—	*HEPHL1*; *PRKDC*; *ZNF44*	—	*BCORL1*; *FAM47A*; *KCNE1L*; *MAGEA6*; *ZCCHC12*	—
F2	Female	*GRIN2A*	*FAM83E*; *KIAA1239*; *KIAA1755*; *LAMA5*; *MIA3*	*—*	*—*	*—*
F3^a^	Male	*PPFIBP2*	*C16orf91*; *C9orf79*; *CCDC144NL*; *NHSL1*	*—*	*CCDC22*; *SHROOM2*	[*H2BFM*; *H2BFWT*]
F5	Male		*DLC1*; *TTN*	*—*	*FAM70A*; *FTHL17*; *GPR112*; *PCDH19*; *RBMXL3*; *WDR44*	*—*
F6	Female	*C11orf41*; *NF1*; *SMARCC2*; *ZHX3*^b^	*FAM188B*; *RELN*; *RERE*	*AXL*	*—*	*—*
F7	Female	*UNC80*; *WFDC8*	*MUC16*; *TSC22D1*; *TTN*	*—*	*—*	*—*
F8	Male	*CD244*	*LY75-CD302*; *TTN*; *WDR59*	*—*	*PLXNB3*; *RBBP7*; *SRPX2*	*—*
F9	Male	*PARD3B*	*ABCA13*; *COL6A6*; *GNAS*; *KIAA1462*; *MUC17*; *SRRM2*; *TRPM8*	*—*	*ATP2B3*; *CCDC22*	*—*
F10	Female	*ATP6V1B2*; *SEMA4D*^c^	*C19orf28*; *CDHR1*; *DNAH10*; *MACF1*	*—*	*—*	*—*
F11	Male	*—*	*REST*	*—*	*CITED1*; *MXRA5*; *NR0B1*	*—*
F12	Female	*—*	*FRG1B*; *TTN*; *ZNF451*	*—*	*—*	*—*
F13	Male	*—*	*FRAS1*; *SPTBN5*; *TPO*	*—*	*ALG13*; *DDX26B*; *MAP7D3*; *TLR7*	*—*
F14	Female	*KCTD8*; *STX12*	*ADNP*; *ANO7*; *CENPF*; *TDRD6*	*—*	*—*	[*GPM6B*; *OFD1*]
F15	Female	*DOCK1*	*ABLIM3*; *VCAN*	*—*	*—*	*—*
F16^a^	Male	*PPFIBP2*	*C16orf91*; *C9orf79*; *CCDC144NL*; *NHSL1*	*—*	*SHROOM2*	*—*
F17	Female	*—*	*ABCA3*; *AKAP11*; *DEPDC1*; *PAFAH2*; *POM121C*	*—*	*—*	*—*
F18	Male	*ABCB9*; *FAM3D*	*PCCB*; *TTN*; *ZFHX3*	*—*	*CXorf57*; *DUSP21*; *F9*; *FOXR2*; *HS6ST2*; *NKAP*; *RBMX2*	*—*
F19^e^	Male	*DNAJC13*^b^; *NLRP1*; *PARD3B*	*AHNAK2*; *C20orf90*; *CD163L1*; *DNAH1*; *DNAH5*; *DNAH6*; *FSTL4*; *PHLPP2*	*ADAD2*; *PCNT*	*COL4A6*; *GYG2*; *PNMA3*; *SATL1*; *SHROOM2*	[*SSX3*; *SSX4*; *SSX4B*]
F20	Male	*COL2A1*	*CHD7*; *EPB41L2*; *GPR98*; *VPS13D*	*—*	*FAM58A*; *MTCP1NB*; *PLXNA3*; *SLC10A3*	*—*
F21	Male	*—*	*CACNA1H*; *PKHD1*	*KIF26A*	*ARMCX2*; *EDA2R*; *HTATSF1*; *MAP7D3*; *MTMR8*; *MXRA5*	*—*
F22	Male	*TACR2*	*DECR1*; *DUOXA1*; *NEB*; *VPS13C*	*PCDHB7*	*MAP7D3*	*—*
F23	Male	*FGFR3*	*C1orf129*; *SHANK2*; *TTN*	*GFM2*	*MAP3K15*; *MAP7D3*	*—*
F25	Male	*PNLIPRP1*; *SMARCC1*	*HSPG2*; *IQGAP3*	*—*	*BCOR*; *RAB40A*; *USP26*	*—*
F26	Male	*KDM5B*; *STAU2*	*GNRHR2*	*—*	*HTATSF1*; *MTMR1*; *PIR*	*—*
F27^d^	Female	*C2orf40*; *INSC*	*—*	*—*	*—*	*—*
F28	Female	*PPP6R1*	*CYP24A1*; *KIAA1109*; *KIAA1609*; *SLC39A11*	*—*	*—*	*—*
F29	Female	*—*	*ABCA13*; *MCF2L2*; *NLRP12; POM121C; TTN*; *ZNF831*	*TTN*	*—*	*—*
F31	Female	*FMNL3*	*FAH*	*—*	*—*	*—*
F32	Female	—	—	*—*	*—*	*—*
F33^d^	Female	*SEC31B*; *EGFL6*^b^	*AGRN*; *NUDT19*	*—*	*—*	*—*

Square brackets contain genes in a single CNV. Variant information is detailed in the Supplementary Material, Appendix for the *de novo* SNPs and indels (Supplementary Material, Table S2), inherited recessive and X-linked SNPs and indels (Supplementary Material, Table S3) and CNVs (Supplementary Material, Table S4 and Supplementary Material, Fig. S4). ‘Possibly causal’ genes are indicated in orange and ‘highly likely to be causal’ genes in red.

^a^Monozygotic twins.

^b^Synonymous *de novo* variant.

^c^We looked for inherited, rare, functional, ‘second hit’ variants in genes in which we found *de novo* mutations and found only one: a 9:92006277C>G heterozygous, maternally inherited missense variant in *SEMA4D* in F10.

^d^Siblings.

^e^This fetus is of Indian ancestry, whereas the majority of the cohort is of European ancestry. This is likely to explain why there are so many apparently rare inherited candidate variants in this case.

The expected percentage of *de novo* variants in coding or splicing sequence that are synonymous is 29% (12), however, we observed that only three (9%) of the 33 validated independent *de novo* variants were synonymous, with 26 being non-synonymous, three nonsense and one in a splice site. Thus the proportion of validated *de novo* mutations that are predicted to have a functional consequence of the encoded protein is significantly enriched over what would be expected by chance (*P* = 0.007), suggesting that an appreciable subset of these functional mutations is likely to be pathogenic. Of the non-synonymous variants, 10 (38.5%) are predicted to be probably damaging by Polyphen, 13 (50%) are predicted to be deleterious by sorting intolerant from tolerant, and seven (26.9%) are predicted to be damaging by both methods (Supplementary Material, Table S2). We found suggestive evidence of mosaicism for two of the *de novo* mutations: c.313G>C (p.105E>Q) in *PARD3B* (ENST00000349953) in F9, and c.2921G>T (p. 974C>F) in *SEC31B* (MIM 610258, ENST00000370345) in F33 (Supplementary Material, Table S2).

Two of the *de novo* missense variants are highly likely to be pathogenic. In F23, a male fetus with features consistent with lethal skeletal dysplasia, we found c.1118A>G (p.373Y>C) in *FGFR3* (MIM 134934 (http://www.omim.org/), ENST00000440486 (http://www.ensembl.org/)). FGFR3 is a negative regulator of bone growth, missense mutations in which cause a wide range of skeletal dysplasias. p.373Y>C is known to cause thanatophoric dysplasia (13), giving high confidence that this is the causative mutation in F23.

In F20, a male fetus with increased nuchal translucency (>3.5 mm), tricuspid regurgitation and abnormal legs and feet with an extended posture and bilateral talipes equinovarus anomaly, we found c.3490G>T (p.1164G>C) in *COL2A1* (MIM 120140, ENST00000380518). *COL2A1* mutations can cause type II collagenopathies, some of which include heart and limb defects (14,15). Importantly, p.1164G>C is a glycine to non-serine substitution in the triple helical domain of COL2A1, which is a particularly damaging class of mutations (16), although p.1164G>C has not previously been reported.

We found two possibly pathogenic *de novo* missense variants in F6, a female fetus with abdominal situs inversus, cardiac malposition of the great arteries and multiple ventricular septal defects. These features are consistent with Ivemark's syndrome (MIM 208530), the molecular basis of which is unknown. First, we found c. 2747G>A (p.916R>Q) in *NF1* (MIM 613113, ENST00000456735). Variants in *NF1* can be associated with congenital heart defects, and knocking down either zebrafish orthologue causes cardiovascular defects (17,18). Furthermore, substitution of this particular amino acid has been previously proposed to be pathogenic (19). Second, we found c.1555C>T (p.519R>*) in *SMARCC2* (MIM 601734, ENST00000267064). This encodes a SWItch/Sucrose NonFermentable-related chromatin regulator. Variants within several genes that encode components of the same protein complex can cause developmental disorders (20,21).

Two of the unrelated fetuses had *de novo* missense mutations in *PARD3B*. F9, a male fetus with a complex brain malformation and unilateral talipes equinovarus had the *PARD3B* mutation c.313G>C (p.105E>Q). F19, a male with an atrial septal defect, esophageal atresia and a unilateral facial cleft had the mutation c.731G>A (p.244R>Q). The likelihood of two functional *de novo* mutations in a gene of the size of *PARD3B* occurring by chance in unrelated probands in a cohort of this size is small (*P* = 3.1 × 10^−6^, which does not reach the Bonferroni-corrected significance threshold for testing of all genes of *P* = 2.5 × 10^−6^). *De novo PARD3B* mutations have not been reported in other larger sequencing studies suggesting that *PARD3B* does not have an unusually high mutation rate (9,11). *PARD3B* encodes partitioning defective 3 homolog B (Par3b), which is involved in cell polarization (22). It has a paralogue, *PARD3,* which has a role in various developmental processes including neurogenesis (23). Homozygous mouse knockouts for *Par3* are embryonic lethal and have growth retardation, heart and brain defects and short tails (24), and zebrafish Pard3 knockdowns have hydrocephalus (23). The overlap between phenotypes resulting from knockdown of *PARD3* and the phenotypes in F9 and F19 is interesting, however we judged that the current knowledge of the function of *PARD3B* is insufficient to categorize the mutations identified in our cohort as being possibly causal.

*De novo* variants in genes known to be involved in developmental disease were not necessarily classified as possibly causal, where the phenotype of the fetus did not overlap sufficiently with previously reported phenotypes. For example, the *de novo* missense mutation c.4354C>T (p.1452R>C) in *GRIN2A* (MIM 138253, ENST00000461292) was found in F2, a female with atrioventricular septal defect (AVSD), hepatic dysfunction, polydactyly, panhypopituitarism and brain injury. *GRIN2A* mutations can cause seizures and intellectual disability, and are highly unlikely to be the cause of the multiple structural malformations seen in F2 (25). Supporting this assertion is the fact that this individual had an older sibling with a similar phenotype, making *de novo* variants an unlikely cause of disease.

### Inherited recessive and X-linked SNVs and indels

We detected a mean of 21 444 high-quality coding SNVs and indels per individual (Supplementary Material, Table S1). We identified potentially relevant inherited recessive and X-linked variants (SNVs and indels) by filtering for rare (minor allele frequency <1%), functional, hemizygous, homozygous or compound heterozygous mutations. This identified a mean of 5.3 candidate genes per fetus (range of 0–15) with a total of 139 different candidate genes across the 30 fetuses, containing 269 rare functional variants. Of these variants, 262 are missense, four are frameshift and three are nonsense (Table 1 and Supplementary Material, Table S3).

Inherited variants in five of the fetuses are possibly causal. These mutations have been verified by Sanger sequencing of whole genome amplified genomic DNA (data not shown). In F1, a male fetus with multiple abnormalities including limb defects, craniofacial defects, anogenital defects, heart defects, a tracheal esophageal fistula and renal agenesis, we found the compound heterozygous mutations c.9598C>T (p.3200P>S) and c.1420G>T (p.474V>F) in *PRKDC* (MIM 600899, ENST00000338368). *PRKDC* encodes DNA-PKcs, which, in complex with Ku, is required for the DNA double-strand break repair mechanism non-homologous end-joining. In humans, *PRKDC* mutations can cause severe combined immunodeficiency due to defective V(D)J recombination, and severe cases can also have abnormalities of the brain, face, limbs and anogenital organs (26).

In F5 who had cardiac truncus arteriosus, type B interruption of the aortic arch and pyloric stenosis, we found the compound heterozygous mutations c.2189G>A (p.730R>Q) and c.721C>G (p.241P>A) in *DLC1* (MIM 604258, ENST00000276297). Homozygous *DLC1* knockout mice are embryonic lethal with deformities of brain and heart (27). In F6, whose phenotype has been described, we found the compound heterozygous mutations c.4264G>A (p.1422V>M) and c.3686G>A (p.1229R>Q) in *RERE* (MIM 605226, ENST00000337907). Homozygous mouse knockouts develop asymmetrically and have cardiovascular defects, while homozygous zebrafish mutants have various defects including abnormal fins and brains (28–30). In total we have identified two genes with *de novo* variants and one gene with inherited variants that could possibly account for the phenotype in F6. It is not possible to say which is most likely to be causative, as none of the candidate genes are known to harbor variants that cause the exact phenotype reported here. One possibility is that multiple variants contribute to this multisystemic phenotype, as has been reported in other exome sequencing studies of rare disease (5).

F10 had fetal akinesia syndrome probably caused by neuroaxonal dystrophy. We found the compound heterozygous mutations c.5323G>A (p.1775E>K) and c.8626A>G (p.2876I>V) in *MACF1* (MIM 608271, ENST00000372925), which encodes cytoskeletal protein microtubule-actin cross-linking factor 1. Knockout of the mouse orthologue causes defects in axonal extension (31). Finally, F13 had multiple abnormalities including a multicystic-dysplastic kidney, distorted ribs and spine, brain defects and bilateral talipes equinovarus. Here we noted the compound heterozygous missense mutations c.1918C>T (p.640R>C) and c.5205C>A (p.1735H>Q) in *FRAS1* (MIM 607830, ENST00000264895), in which mutations are associated with Fraser syndrome (MIM 219000). FRAS1 has a role in renal development (32), and knockout mice have severely defective kidney development, along with syndactyly (33). Homozygous zebrafish mutants have malformed fins and pharyngeal pouches, suggesting a role for FRAS1 in skeletal development (34).

F19 has a high number of inherited, apparently rare variants (Table 1). F19 is of Indian ancestry, whereas the majority of the cohort is of European ancestry. It is likely therefore that a subset of the apparently rare variants that we have identified in F19 are in fact common in this population, but we have not been able to identify them as such due to an underrepresentation of individuals of Indian ancestry in the databases we used to filter the variants.

### CNVs

CNVs from the exome data were denoted using the CoNVex program. We identified three rare, high-quality CNVs (one deletion and two duplications) under *de novo*, inherited recessive or X linked models (Table 1 and Supplementary Material, Table S4 and Supplementary Material, Fig. S4).

One of these was the *de novo* 21 kb deletion g.13770686_13791294del on Xp22.2 found in F14, a female fetus with ventriculomegaly and agenesis of the corpus callosum, This CNV removes most of the gene *OFD1* (MIM 300170). Mutations in *OFD1* cause orofaciodigital syndrome 1 (MIM 311200), which in 40% of cases includes central nervous system anomalies, including the absence of the corpus callosum (35). This deletion has been confirmed by aCGH, is highly likely to be pathogenic, and has a low (<1%) recurrence risk.

## DISCUSSION

In this study, we performed exome sequencing of 30 fetuses or newborns (along with their parents) with diverse congenital structural abnormalities identified by fetal (prenatal) ultrasound. We identified an average of one candidate gene with a *de novo* functional variant and five candidate genes with inherited functional variants per fetus. Variants that are highly likely to be causal were noted in three cases (10%). Variants that are possibly causal but require further confirmatory genetic and functional studies were noted in a further five cases (17%).

Proof of principle of prenatal NGS for diagnosis of aneuploidy and chromosomal rearrangements has been established (6,7). Subsequently, four fetuses were included in a cohort of 250 patients with Mendelian disorders who were exome sequenced, of which one was successfully diagnosed (5). There are other cases in which postnatal exome sequencing of cohorts with specific diseases first manifesting in the prenatal period have yielded diagnoses (36,37). Our study of a cohort of 30 fetuses with diverse abnormalities has allowed us to estimate the current diagnostic yield of exome sequencing for fetal abnormalities as being broadly equivalent to aCGH, which is ∼10% (2–4). However, due to the small cohort size our estimate of diagnostic yield has a broad CI. While some of our samples were obtained from neonates subsequent to live birth, the abnormalities of the entire cohort were first identified at the prenatal stage, so our estimated diagnostic yield is equivalent to what would be expected for entirely prenatal testing. Larger studies would not only provide a more accurate diagnostic rate, but would allow for additional interesting analyses such as stratification of the cohort on the basis of phenotypic severity, which could aid in identification of pathogenic variants.

The primary challenge faced in this study that is shared across all such studies was interpreting the clinical significance of the candidate mutations we identified. There is therefore considerable potential for increase of the diagnostic yield once understanding of the genetic architecture of developmental disorders has improved. However, an additional factor here is that for most genes where disruptive genetic variants can cause developmental disorders, our understanding of the phenotypic consequences of this variation is limited to postnatal observations that are often not detectable by prenatal ultrasound investigations (e.g. intellectual disability in the absence of structural brain malformations). This could explain why our diagnostic yield is lower than that of similar studies of postnatal disorders (5). Moreover, given that postnatal observations are inherently subject to survival bias, it may be that in the prenatal setting more severe phenotypes can be observed for the same variants (38).

The *de novo* occurrence of the three clearly pathogenic variants identified in this prenatal study could have enabled the parents to be counseled that the risk of recurrence in subsequent pregnancies is low, and highlights the value to families of receiving a clear genetic diagnosis. In this study, none of the results from the exome sequencing were relayed to patients. Our finding that most currently diagnostic variants from exome sequencing arise *de novo* is in agreement with recent studies of patients with intellectual disability, many of whom also have congenital structural abnormalities (9,11). Interestingly, only one of the three variants that are highly likely to be causal would have been detected by aCGH alone (the CNV overlapping *OFD1* in F14), and only one could have been suspected as a candidate gene from the ultrasound findings alone (*FGFR3* variants in F23 with thanataphoric dysplasia).

Clinical implementation of exome sequencing for prenatal diagnosis of structural anomalies identified by ultrasound promises to improve management of pregnancy and enable more informative counseling to parents. However, it also poses several challenges. First, the analytical strategy we adopted was labor intensive and not scalable. Clinical implementation would require the development of large-scale and rapid analytical and interpretation pipelines, as well as rigorous health economic assessments. Second, to facilitate interpretation of prenatal variation, it will be necessary to develop a vastly more detailed knowledge base on the genetic causes of prenatal developmental disorders. Finally, the sequencing data in this study were not produced within a timeframe that would have allowed the parents to take it into account when making a decision about the outcome of the pregnancy being tested. This would of course be the ideal scenario, and feasibility in principle has been demonstrated (6).

In conclusion, our study shows that exome sequencing is a promising method with which to identify genetic variants that cause structural fetal abnormalities and thereby improve the clinical management of pregnancies and better inform the reproductive decisions of affected families. The challenges to clinical implementation are considerable, but surmountable, and we envisage that exome sequencing, initially in a clinical research context, will be an important addition to prenatal genetic diagnostics in the near future.

## MATERIALS AND METHODS

### Cohort

The National Research Ethics Service Committee (West Midlands—Staffordshire, UK) approved this study (REC reference 09/H1203/74). We recruited a cohort of 33 fetuses and their non-consanguineous parents seen for prenatal diagnosis at the Fetal Medicine Centre Birmingham Women's Foundation Trust, UK. This is a subgroup (12%) of a larger cohort described previously (2). The median maternal age at diagnosis was 31 years (range 18–39). We included women who had a fetus with a structural anomaly suspected at their routine ultrasound scan at 11–14 weeks or 18–20 weeks gestation. The women were subsequently rescanned at a tertiary referral center and the diagnoses confirmed. The median gestational age at confirmation of diagnosis was 21 weeks (range 11–35).

DNA of affected fetuses or neonates was obtained from various sources. Where a pregnancy was terminated or miscarried, fetal DNA was most commonly collected from placental tissue. Where a live birth occurred, neonatal DNA was usually collected from cord blood at delivery (Supplementary Material, Appendix and Table 2). Parental DNA was obtained from venous blood. In this article, the participants are identified by their trio number (1–33) prefaced by F for the fetus, M for the mother and P for the father. There are two exceptions to this, as the cohort includes two sets of related fetuses. F3 and F16 are monozygotic twins; therefore the parents of F16 are M3 and P3. F27 and F33 are siblings; therefore the parents of F33 are M27 and P27. F2 has an older sibling with a similar phenotype, who is not included in this study. The remaining fetuses are sporadic cases, and none of the parents had phenotypic abnormalities that were likely to be related to that of the fetuses.

**Table 2.** Sampled tissue and outcomes

Pregnancy outcome	Number (and percent of cohort)	Sampled tissue	Number (and percent of cohort)
Termination or miscarriage	19 (63%)	Placenta	12 (40%)
		Blood	4 (13%)
		Chorionic villus sample	2 (7%)
		Other tissues (fetal liver/lung)	1 (3%)
Live birth	11 (37%)	Cord blood	6 (20%)
		Chorionic villus sample	2 (7%)
		Placenta (postnatally)	1 (3%)
		Cultured amniocytes	1 (3%)
		Postnatal venous blood	1 (3%)

Prior to inclusion in this study the G-band karyotypes of all cases except F33 were confirmed as normal, and a low-resolution targeted 1 Mb BAC array (BlueGnome) did not demonstrate any likely pathological CNVs. For F33, a combination of quantitative fluorescent PCR and multiplex ligation-dependent probe amplification revealed no aneuploidy, or deletions or duplications in the subtelomeric regions. For the 95 samples, we obtained a mean of 3 μg DNA, but 12 samples had less than the required 0.2 μg DNA for exome sequencing. We attempted to sequence all samples, but exome sequencing failed due to insufficient DNA in trios 4, 24 and 30, leaving a total cohort of 26 trios and two quads (couple with two affected fetuses) (91%, 86 samples).

**Figure 1.**
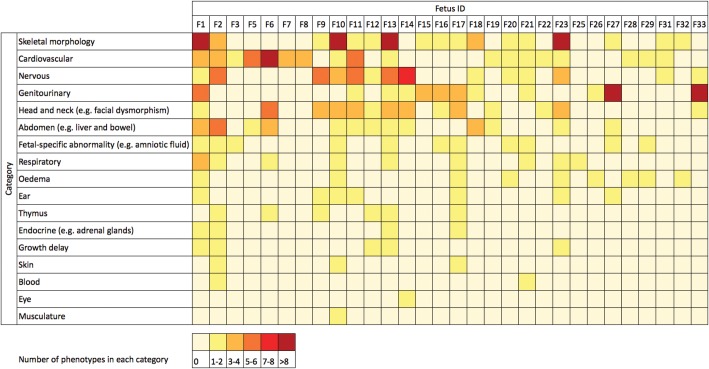
Matrix of phenotypes in the cohort. For each fetus (F1–F33), the color indicates the number of observed phenotypes that are in each category of phenotypes. For example, F1 has more than eight separate abnormalities of skeletal morphology. The categories are modified higher order HPO terms, and the data come from ultrasound scans, postmortem reports or pediatric follow-up. The phenotype of each fetus is detailed in the Supplementary Material, Appendix.

The fetuses and neonates had a wide range of structural abnormalities. In addition to those identified by detailed ultrasound scan and investigation in a tertiary Fetal Medicine Centre, phenotypic information was collected for a subset of cases from postmortem reports by perinatal pathologists (*n* = 12), or pediatric follow up (*n* = 7). Phenotype data were converted to Human Phenotype Ontology (HPO) terms (Fig. 1 and Supplementary Material, Appendix) (39).

### Exome sequencing and candidate variant identification

For detailed methodology see Supplementary Material, Appendix. In brief, DNA samples were exome sequenced using a SureSelect All Exon capture kit (Agilent, Wokingham, UK) followed by paired-end sequencing (75 bp reads) on the HiSeq platform (Illumina, Saffron Walden, UK). Reads were mapped to the reference human genome GRCh37_hs37d5. Duplicate reads were removed and reads were realigned around potential indels. We called variants using SAMtools, GATK and Dindel. The EGA ID for the exome sequencing data is EGAS00001000167 (https://www.ebi.ac.uk/ega/).

To identify *de novo* mutations, we used *De Novo* Gear pipeline version 0.6.2 (40). We considered rare, high-quality variants in protein-coding exons. To identify inherited recessive and X-linked SNVs and indels, we first merged the VCFs from the three variant callers for each individual, and then we identified genes harboring rare, high-quality, functional variants (predicted protein consequences were essential splice site, stop gained, frameshift coding, non-synonymous, stop lost) under the different plausible modes of inheritance (recessive or X-linked). To discover CNVs from the exome data, we used CoNVex (ftp://ftp.sanger.ac.uk/pub/users/pv1/CoNVex/). We filtered out CNVs that were low confidence, overlapped common CNVs, did not overlap protein-coding genes or did not fit with the expected mode of inheritance (*de novo*, inherited recessive or X-linked).

### Classification of variants

**Figure 2.**
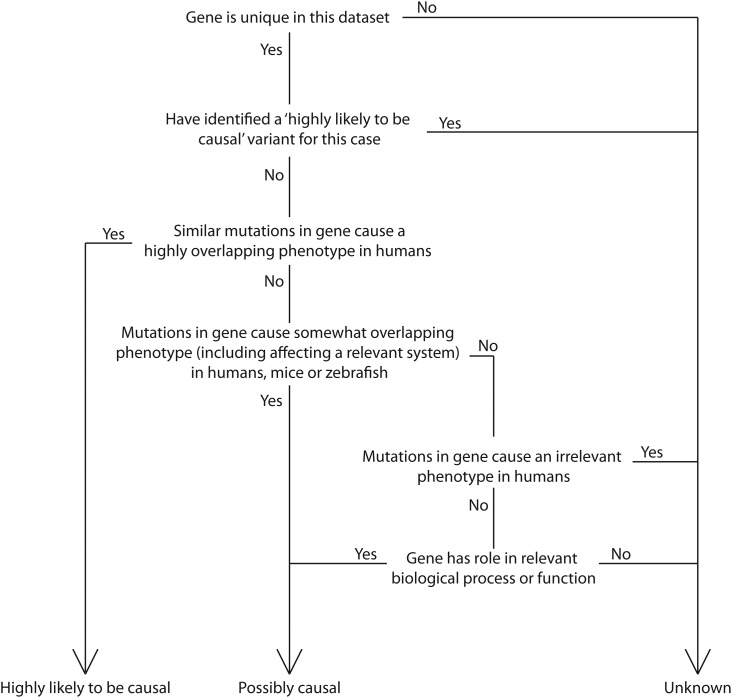
Decision tree for prioritizing candidate genes into three categories. Data from OMIM, DDG2P, BioGPS, NHGRI GWAS catalog, IKMC, ZFIN and PubMed were used, where available.

To classify the variants we annotated each candidate gene with functional information (where available) from OMIM (http://www.omim.org/), DDG2P (http://decipher.sanger.ac.uk/ddd/ddd_genes), BioGPS (biogps.org), NHGRI GWAS catalog (http://www.genome.gov/gwastudies/), IKMC (http://www.knockoutmouse.org/), ZFIN (http://zfin.org/) and PubMed (http://www.ncbi.nlm.nih.gov/pubmed). We used a decision tree to classify each variant as being highly likely to be causal, possibly causal but requires further genetic or functional confirmatory studies, or unknown (Fig. 2).

## SUPPLEMENTARY MATERIAL

Supplementary Material is available at *HMG* online.

## FUNDING

This work was supported by the Wellcome Trust (WT098051); and SPARKS—The Children's Medical Research Charity (09RTF07 to S.C.H.). Funding to pay the Open Access publication charges for this article was provided by the Wellcome Trust (WT098051).

## Supplementary Material

Supplementary Data
