# Supplementary material for: Exome sequencing improves genetic diagnosis of structural fetal abnormalities revealed by ultrasound
Source: Hum Mol Genet. 2014 Jan 29;23(12):3269–77. doi: 10.1093/hmg/ddu038 (PMC4030780; doi:10.1093/hmg/ddu038)
Supplement: Supplementary Data [file supp_23_12_3269__index.html]

Exome sequencing improves genetic diagnosis of structural fetal abnormalities revealed by ultrasound — Exome sequencing improves genetic diagnosis of structural fetal abnormalities revealed by ultrasound — Exome sequencing improves genetic diagnosis of structural fetal abnormalities revealed by ultrasound — Supplementary Data 

# Exome sequencing improves genetic diagnosis of structural fetal abnormalities revealed by ultrasound

## Supplementary Data

Supplementary Data

**Files in this Data Supplement:**

- Supplementary Data - Pdf file
