# Supplementary material for: Exome sequencing improves genetic diagnosis of structural fetal abnormalities revealed by ultrasound
Source: Hum Mol Genet. 2014 Jan 29;23(12):3269–77. doi: 10.1093/hmg/ddu038 (PMC4030780; doi:10.1093/hmg/ddu038)
Supplement: Supplementary Data [file supp_ddu038_ddu038supp.pdf]

Supplementary Appendix

Exome sequencing improves genetic diagnosis of structural fetal abnormalities revealed by ultrasound

Keren J. Carss[1], Sarah C. Hillman[2], Vijaya Parthiban[1], Dominic J. McMullan[3], Eamonn R. Maher[2], Mark D. Kilby[2,4]*, Matthew E. Hurles[1]

1) Wellcome Trust Sanger Institute, Wellcome Trust Genome Campus, Hinxton, Cambridgeshire, CB10 1SA, UK.
2) School of Clinical & Experimental Medicine (Birmingham Centre for Women's and Children's Health), College of Medical & Dental Sciences, University of Birmingham, Edgbaston, Birmingham, B15 2TT, UK.
3) West Midlands Regional Genetics Laboratory, Birmingham Women's NHS Trust, Edgbaston, Birmingham, B15 2TG, UK.
4) Fetal Medicine Centre, Birmingham Women's Foundation Trust, Edgbaston, Birmingham, B15 2TG, UK.

* To whom correspondence should be addressed: email m.d.kilby@bham.ac.uk; telephone +44-121-627-2778; fax +44-121-623 6875; School of Clinical & Experimental Medicine (Birmingham Centre for Women's and Children's Health), College of Medical & Dental Sciences, University of Birmingham, Edgbaston, Birmingham, B15 2TT, UK.

Clinical Details of Fetuses Including HPO Terms

F1

A 39 year old woman presented at 22 weeks gestation to a tertiary referral centre with multiple fetal anomalies detected on ultrasound scan (USS). These included a cardiac ventricular septal defect (VSD), mild ventriculomegaly of the brain, an absent right kidney, and anhydramnios. The couple opted for a termination of pregnancy. Post-mortem examination revealed a male fetus whose measurements were less than expected for 22 weeks gestation (body weight 206 grams (normal = 473±63 grams), crown heel length 21 cm (normal = 27·8±1·6 cm)). External anomalies included dysmorphic facies, ulno-radial agenesis, oligodactyly, transverse reduction of the right lower limb, anal atresia, and ambiguous genitalia. Internal anomalies included a cardiac double outlet right ventricle, VSD, small left cardiac ventricle, tracheal oesophageal fistulae, and renal agenesis. No central nervous system (CNS) post-mortem examination was performed, as brain tissue was not available. Karyotype was that of a normal male (46, XY). Microarray testing was normal both on the 1Mb BAC targeted array platform (BlueGnome Cambridge) and the higher resolution whole genome 2·7M array (Affymetrix). The source of DNA from F1 for sequencing was a fetal blood sample.

Edema (HP:0000969); Abnormality of the amniotic fluid (HP:0001560); Anal atresia (HP:0002023); Esophageal atresia (HP:0002032); Abnormality of the rectum (HP:0002034); Abnormality of the liver (HP:0001392); Ventricular septal defect (HP:0001629); Abnormality of the left ventricle (HP:0001711); Double outlet right ventricle (HP:0001719); Low-set ears (HP:0000369); Abnormality of adrenal morphology (HP:0011732); Ambiguous genitalia (HP:0000062); Abnormality of the testis (HP:0000035); Aplasia of the bladder (HP:0010477); Unilateral renal agenesis (HP:0000122); Ureteral agenesis/dysgenesis (HP:0008631); Oral cleft (HP:0000202); Upslanted palpebral fissure (HP:0000582); Mild fetal ventriculomegaly (HP:0010952); Pulmonary hypoplasia (HP:0002089); Abnormal lung lobation (HP:0002101); Tracheoesophageal fistula (HP:0002575); Oligodactyly (hands) (HP:0001180); Abnormality of the lower limb (HP:0002814); Abnormality of the ilium (HP:0002867); Rib fusion (HP:0000902); Supernumerary ribs (HP:0005815); Thoracic hypoplasia (HP:0005257); Hemivertebrae (HP:0002937); Block vertebrae (HP:0003305); Abnormality of the vertebrae (HP:0003468); Unilateral radial aplasia (HP:0011908); Aplasia/Hypoplasia of the ulna (HP:0006495); Intrauterine growth retardation (HP:0001511).

F2

A 32 year old woman presented at 21 weeks gestation to a tertiary referral centre with multiple fetal anomalies present on USS including a VSD, polydactyly, mild hyperechogenic bowel, and oligohydramnios. A baby girl was born by elective Caesarean section with Apgar scores of seven at one minute following delivery, and nine at five minutes following delivery. In the postnatal period her cardiac anomaly was confirmed as an atrioventricular septal defect (AVSD). She was also diagnosed with hepatic dysfunction and panhypopituitarism. She was admitted to hospital for potential cardiac surgery but had signs of septicemia and was admitted to intensive care. Magnetic resonance imaging (MRI) showed extensive brain injury. She died at five months of age, at which time her measurements were <3rd centile for her age. Post-mortem examination showed internal anomalies including a partial cardiac AVSD, an enlarged liver, hypoplastic adrenal glands, and an atrophic pituitary gland. Cytogenetics showed a normal female karyotype (46, XX). Microarray testing was normal both on the 1Mb BAC targeted array platform (BlueGnome Cambridge) and the higher resolution ISCA v2·0 60K array (Bluegnome). This couple also had an older daughter with a similar phenotype, making inherited mutations a more likely cause of this phenotype than *de novo* mutations. The source of DNA from F2 for sequencing was cord blood at delivery.

Arterial thrombosis (HP:0004420); Oligohydramnios (HP:0001562); Echogenic fetal bowel (HP:0010943); Bile duct proliferation (HP:0001408); Malformation of the hepatic ductal plate (HP:0006563); Hepatic fibrosis (HP:0001395); Hepatomegaly (HP:0002240); Abnormality of the liver (HP:0001392); Defect in the atrial septum (HP:0001631); Atrioventricular canal defect (HP:0006695); Persistent left superior vena cava (HP:0005301); Arteriovenous malformation (HP:0100026); Adrenal hypoplasia (HP:0000835); Panhypopituitarism (HP:0000871); Abnormality of the thymus (HP:0000777); Skin rash (HP:0000988); Diffuse brain atrophy (HP:0002283); Cerebellar hemorrhage (HP:0011695); Abnormality of the cerebral cortex (HP:0002538); Hypoplasia of the frontal lobes (HP:0007333); Abnormality of the midbrain (HP:0002418); Respiratory tract infection (HP:0011947); Postaxial polydactyly (hands) (HP:0001162); Postaxial polydactyly of foot (HP:0001830); Small feet (HP:0001764); Small hands (HP:0200055); Growth delay (HP:0001510).

F3 and F16

A 28 year old woman presented to a tertiary referral centre at 17 weeks and six days gestation with monochorionic diamniotic twins (F3 and F16). F3 had an isolated cardiac VSD on USS. F16 had multiple anomalies on USS including possible sacral hypoplasia, abnormal left and right kidneys, and a dilated and tense bladder (probably due to a lower urethral tract obstruction). There were probable talipes and the head was lemon shaped with the presence of nuchal thickening. The couple decided to undergo a selective termination of pregnancy of F16 due to the USS features. F3 subsequently miscarried. No post-mortem examination was performed. Cytogenetics showed two normal male 46, XY karyotypes. Microarray testing was normal both on the 1Mb BAC targeted array platform (BlueGnome, Cambridge) and the higher resolution ISCA v2·0 60K array (Bluegnome). The source of DNA from F3 and F16 for sequencing was a tissue sample.

F3: Oligohydramnios (HP:0001562); Ventricular septal defect (HP:0001629)

F16: Abnormal insertion of umbilical cord (HP:0011418); Thickened nuchal skin fold (HP:0000474); Fetal megacystis (HP:0010956); Abnormality of the kidney (HP:0000077); Abnormality of the lower urinary tract (HP:0010936); Abnormality of calvarial morphology (HP:0002648); Talipes (HP:0001883); Hypoplastic sacrum (HP:0004590)

F5

A 31 year old woman presented to a tertiary referral centre at 21 weeks and five days gestation with a fetal cardiac anomaly on USS (probable aortic atresia, a VSD and absent pulmonary valve syndrome). She underwent an emergency Caesarean section at term, and a baby boy was born. Postnatally the cardiac defect was confirmed as a truncus arteriosus and type B interrupted aortic arch. Follow up at seven months of age reported that he had several cardiac operations and had a surgical repair of his pyloric stenosis. He was a healthy, alert and responsive child with development entirely appropriate to his age. Karyotyping had shown a normal male 46, XY karyotype. The 1Mb BAC array result showed a duplication Xp22·32p22·31 (RP11-60N3->RP11-769N24). This result was confirmed, and the

breakpoints were refined, using an Affymetrix whole-genome 2·7M array. There were two separate duplications at Xp22·32p22·33 separated by ~412 kb. The first was 465 kb long and appears to be the more significant as it disrupts the *NLGN4* gene. The second duplication was 201 kb with no HGNC mapped genes and most likely represents copy number polymorphism due to the amount of similar cases in the Database of Genomic Variation. Although not linked to cardiac anomalies *NLGN4* is linked to autism. X inactivation studies were inconclusive. This was reported to the parents as a variant of unknown significance. This result has previously been described. (1) The source of DNA from F5 for sequencing was cord blood at delivery.

Pyloric stenosis (HP:0002021); Ventricular septal defect (HP:0001629); Absence of the pulmonary valve (HP:0005134); Truncus arteriosus (HP:0001660); Interrupted aortic arch hype B (HP:0011613); Abnormality of the aorta (HP:0001679)

F6

A 34 year old woman presented to a tertiary referral centre at 22 weeks gestation with a complicated fetal cardiac defect on USS (levocardia with abdominal situs inversus (stomach on the right), a complete AVSD with malposed great arteries, multiple VSDs, and possible right atrial isomerism). The couple opted for a termination of pregnancy. A post-mortem examination showed a 23 week gestation fetus with appropriate measurements. A cardiac defect was confirmed showing malposition of the great arteries, transposition of the pulmonary veins, a double outlet right ventricle, an AVSD, and right atrial isomerism. There were bilateral trilobed lungs and a symmetrical liver. The gallbladder, stomach, duodenum, and pancreas were on the right side. There was no spleen present and the thymus was small. Some of these symptoms are consistent with Ivemark syndrome, the genetic cause of which is unknown. Cytogenetics showed a normal female 46, XX karyotype. Microarray testing was normal both on the 1Mb BAC targeted array platform (BlueGnome, Cambridge) and the higher resolution ISCA v2·0 60K array (Bluegnome). The source of DNA from F6 for sequencing was a tissue sample.

Abdominal situs inversus (HP:0003363); Asplenia (HP:0001746); Abnormality of the liver (HP:0001392); Right atrial isomerism (HP:0011536); Abnormality of the left ventricle (HP:0001711); Abnormality of the coronary sinus

(HP:0011642); Ventricular septal defect (HP:0001629); Atrioventricular canal defect (HP:0006695); Double outlet right ventricle (HP:0001719); Abnormality of the pulmonary veins (HP:0011718); Right aortic arch (HP:0012020); Persistent left superior vena cava (HP:0005301); Abnormality of the heart (HP:0001627); Small chin (HP:0000331); Flat forehead (HP:0004425); Flat nose (HP:0000457); Micrognathia (HP:0000347); Abnormality of the head (HP:0000234); Hypoplasia of the thymus (HP:0000778); Bilateral trilobed lungs (HP:0011861)

F7

An 18 year old woman presented at 20 weeks and four days gestation to a tertiary referral centre with a complicated fetal cardiac defect on USS (mesocardia with a VSD and a possible over-riding aorta). A female infant was born and the cardiac defect was confirmed as congenitally corrected transposition of great arteries and VSD. She has since undergone two operative procedures. Cytogenetics showed a normal female 46, XX karyotype. Microarray testing was normal both on the 1Mb BAC targeted array platform (BlueGnome Cambridge) and the higher resolution ISCA v2·0 60K (Bluegnome). The source of DNA from F7 for sequencing was cord blood at delivery.

Mesocardia (HP:0011599); Ventricular septal defect (HP:0001629); Congenitally corrected transposition of the great arteries (HP:0011540); Overriding aorta (HP:0002623)

F8

A 21 year old woman presented at 28 weeks and two days gestation to a tertiary referral centre with a complicated fetal cardiac defect on USS (a congenitally corrected transposition of the great arteries, a VSD, mild sub-pulmonary obstruction, and a hypoplastic right valve). The couple opted for a termination of pregnancy. No post-mortem examination was performed. Cytogenetics showed a normal male 46, XY karyotype. Microarray testing was normal both on the 1Mb BAC targeted array platform (BlueGnome Cambridge) and the ISCA v2·0 higher resolution 60K (Bluegnome). The source of DNA from F8 for sequencing was a tissue sample.

Abnormality of the tricuspid valve (HP:0001702); Ventricular septal defect (HP:0001629); Pulmonic stenosis (HP:0001642); Congenitally corrected transposition of the great arteries (HP:0011540)

F9

A 26 year old woman presented to a tertiary referral centre at 20 weeks and five days gestation with fetal anomalies on USS including severe ventriculomegaly bilaterally (>15mm) and a hypoplastic cerebellum. The fetus also appeared to have right-sided talipes. The couple opted for a termination of pregnancy. Post-mortem examination showed an appropriately sized male fetus. Confirmed anomalies included dysmorphic features (an enlarged head, wide sutures/fontanels and low set ears). Post-mortem examination of the CNS showed features consistent with rhombencephalosynapsis and ventriculomegaly. Cytogenetics showed a normal male 46, XY karyotype. Microarray testing was normal both on the 1Mb BAC targeted array platform (BlueGnome Cambridge) and the higher ISCA v2·0 resolution 60K (Bluegnome). The source of DNA from F9 for sequencing was a tissue sample.

Low-set ears (HP:0000369); Megalencephaly (HP:0001355); Macrocephaly (HP:0000256); Frontal bossing (HP:0002007); Widely patent fontanelles and sutures (HP:0004492); Abnormality of the thymus (HP:0000777); Ventriculomegaly (HP:0002119); Abnormality of the cerebral cortex (HP:0002538); Agenesis of cerebellar vermis (HP:0002335); Fusion of the cerebellar hemispheres (HP:0006899); Cerebellar hypoplasia (HP:0001321); Talipes (HP:0001883)

F10

A 35 year old woman presented to a tertiary referral centre at 21 weeks and three days gestation. USS showed multiple fetal anomalies consistent with fetal akinesia syndrome (the legs were extended and both feet showed severe equinous deformity, the arms and hands were in a fixed flexed deformity, and the spine showed a lateral curvature). There was also possible micrognathia present, a right-sided pleural effusion, the stomach was small, and there was evidence of a small cerebellum. The couple opted for a termination of pregnancy. A post-mortem examination showed a female fetus with measurements in keeping with the gestation. The face showed extremely low set ears, down-

slanting palpebral fissures, a narrow nose, and severe micrognathia. Nuchal oedema and bilateral pleural effusions were identified. Musculoskeletal examination showed flexed arms at the elbows and wrists, internal rotation of the hips, hyperextended knees, and bilateral talipes with dislocation of the ankles and extreme loss of muscle bulk. There were pterygia at the shoulders, elbows, and groin. This phenotype resulted from early onset failure of fetal movement. Histological examination of central nervous system samples showed that this akinesia was likely caused by congenital neuroaxonal dystrophy. Cytogenetics showed a normal female 46, XX karyotype. Microarray testing was normal on the 1Mb BAC targeted array platform (BlueGnome Cambridge). The source of DNA from F10 for sequencing was a tissue sample.

Generalized edema (HP:0007430); Edema (HP:0000969); Fetal akinesia sequence (HP:0001989); Abnormality of the stomach (HP:0002577); Abnormality of the diaphragm (HP:0000775); Hypoplastic heart (HP:0001961); Low-set ears (HP:0000369); Narrow nose (HP:0000460); Downslanted palpebral fissures (HP:0000494); Micrognathia (HP:0000347); Fragile skin (HP:0001030); Multiple pterygia (HP:0001040); Amyotrophy (HP:0003202); Astrocytosis (HP:0002446); Abnormality of the microglia (HP:0100708); Cerebellar hypoplasia (HP:0001321); Diffuse axonal swelling (HP:0003405); Pleural effusion (HP:0002202); Pulmonary hypoplasia (HP:0002089); Equinus calcaneus (HP:0008138); Hand clenching (HP:0001188); Broad ribs (HP:0000885); Scoliosis (HP:0002650); Wrist flexion contracture (HP:0001239); Fixed elbow flexion (HP:0006471); Abnormality of the hip joint (HP:0001384); Hyperextensibility of the knee (HP:0010500); Abnormality of the ankles (HP:0003028)

F11

A 24 year old woman presented to a tertiary referral centre at 25 weeks and two days gestation. USS showed multiple fetal anomalies including a cardiac defect (a dilated right heart ventricle with an anomaly of the crux consistent with an AVSD, the great vessels appeared dilated, and the crossing of the aortic arch could not be visualized). The cisterna magnum and the third ventricle of the brain were enlarged, and the long bones appeared shortened. The couple opted for a termination of pregnancy. Post-mortem examination showed a male fetus appropriately grown for 28 weeks gestation. There were dysmorphic facies with low-set ears, a long philtrum, and down-slanting palpebral fissures. There was a midline cleft of the soft palate, a complete AVSD, and agenesis of the corpus callosum. Histologically there were abnormalities of the cerebellum (a dysplastic denate nucleus and glioneuronal heterotopia). The karyotype

was that of a normal male (46, XY). Microarray testing was normal both on the 1Mb BAC targeted array platform (BlueGnome Cambridge) and the higher resolution ISCA v2·0 array (Bluegnome). The source of DNA from F11 for sequencing was a tissue sample.

Intestinal malrotation (HP:0002566); Abnormal liver lobulation (HP:0100752); Right ventricular dilatation (HP:0005133); Hypoplastic heart (HP:0001961); Abnormality of the atrioventricular valves (HP:0006705); Right aortic arch (HP:0012020); Arteriovenous malformation (HP:0100026); Atrioventricular canal defect (HP:0006695); Low-set ears (HP:0000369); Abnormality of the helix (HP:0011039); Dilatation of the renal pelvis (HP:0010946); Renal hypoplasia (HP:0000089); Long philtrum (HP:0000343); Median cleft palate (HP:0009099); Downslanted palpebral fissures (HP:0000494); Heterotopia (HP:0002282); Enlarged cisterna magna (HP:0002280); Dilated third ventricle (HP:0007082); Abnormality of the dentate nucleus (HP:0100321); Agenesis of corpus callosum (HP:0001274); Short long bones (HP:0003026)

F12

A 36 year old woman presented to a tertiary referral centre at 20 weeks and six days gestation. USS showed severe fetal venticulomegaly (>15mm). The parents opted for a termination of pregnancy. A post-mortem examination confirmed ventriculomegaly and hydrocephalus. It additionally revealed right-sided talipes. Cytogenetics showed a normal female 46, XX karyotype. Microarray testing was normal both on the 1Mb BAC targeted array platform (BlueGnome Cambridge) and the higher resolution ISCA v2·0 60K array (Bluegnome). The source of DNA from F12 for sequencing was a tissue sample.

Abnormality of the liver (HP:0001392); Macrocephaly (HP:0000256); Hypoplasia of the thymus (HP:0000778); Abnormality of the thymus (HP:0000777); Ventriculomegaly (HP:0002119); Hydrocephalus (HP:0000238); Talipes (HP:0001883); Intrauterine growth retardation (HP:0001511);

F13

A 38 year old woman presented to a tertiary referral centre at 21 weeks gestation. USS showed multiple fetal anomalies including bilateral multicystic dysplastic kidneys, microcephaly, a banana-shaped cerebellum, hemivertebrae, an Arnold Chiari malformation, nuchal thickening, and possible talipes. The couple opted for a termination of pregnancy. Post-mortem examination showed a male fetus whose measurements were less than expected for 22 weeks gestation. Congenital anomalies included a cystic-dysplastic horseshoe kidney, a high cardiac VSD and a vertebral segmentation defect with distorted ribs and scoliosis of the thoracic/upper lumbar spine. There was a thoraco-lumbar myelomeningocele present and confirmation of the Arnold-Chiari malformation and bilateral talipes. Cytogenetics showed a normal male 46, XY karyotype. Microarray testing was normal on the 1Mb BAC targeted array platform (BlueGnome Cambridge). The source of DNA from F13 for sequencing was a tissue sample.

Oligohydramnios (HP:0001562); Thickened nuchal skin fold (HP:0000474); Anal atresia (HP:0002023); Abnormality of the large intestine (HP:0002250); Ventricular septal defect (HP:0001629); Abnormality of adrenal morphology (HP:0011732); Abnormality of the adrenal glands (HP:0000834); Cystic renal dysplasia (HP:0000800); Microcephaly (HP:0000252); Short neck (HP:0000470); Broad neck (HP:0000475); Abnormality of the thymus (HP:0000777); Cerebral hemorrhage (HP:0001342); Mild fetal ventriculomegaly (HP:0010952); Arnold-Chiari malformation (HP:0002308); Cerebellar malformation (HP:0002438); Occipital myelomeningocele (HP:0007271); Pulmonary hypoplasia (HP:0002089); Abnormal lung lobation (HP:0002101); Talipes (HP:0001883); Misisng ribs (HP:0000921); Thin ribs (HP:0000883); Abnormality of the ribs (HP:0000772); Thoracolumbar scoliosis (HP:0002944); Hemivertebrae (HP:0002937); Vertebral segmentation defect (HP:0003422); Fixed elbow flexion (HP:0006471); Knee flexion contracture (HP:0006380); Intrauterine growth retardation (HP:0001511)

F14

A 34 year old woman presented to a tertiary referral centre at 35 weeks gestation. USS showed severe left sided fetal ventriculomegaly (>15mm) and agenesis of the corpus callosum. The couple opted for a termination of pregnancy. Post-mortem examination showed a female fetus whose weight was on the 18th centile. Post-mortem examination confirmed hydrocephalus, agenesis of the corpus callosum, focal cerebral cortical dysplasia, and possible dysplasia of the denate nucleus and cerebellum. The face showed mild micrognathia and hypertelorism, and the left uterine horn and fallopian tube were absent. Cytogenetics showed a normal female 46, XX karyotype. Microarray testing was

normal on the 1Mb BAC targeted array platform (BlueGnome, Cambridge). The higher resolution ISCA v2·0 60K array (Bluegnome) identified a rare, *de novo* deletion in Xp22.2 (g.13770535_13787331del), which overlaps the protein coding genes *OFD1* and *GPM6B*. This result was reported to the parents. This deletion (with slightly different estimated breakpoints) was also identified from the exome sequencing data using CoNVex. The source of DNA from F14 for sequencing was a fetal blood sample.

Abnormality of the liver (HP:0001392); Hypertelorism (HP:0000316); Hypoplasia of the fallopian tube (HP:0008697); Hypoplasia of the uterus (HP:0000013); Megalencephaly (HP:0001355); Macrocephaly (HP:0000256); Micrognathia (HP:0000347); Wide anterior fontanel (HP:0000260); Ventriculomegaly (HP:0002119); Hydrocephalus (HP:0000238); Abnormal cortical gyration (HP:0002536); Cortical dysplasia (HP:0002539); Gliosis (HP:0002171); Agenesis of corpus callosum (HP:0001274); Hypoplastic olfactory lobes (HP:0006894)

F15

A 31 year old woman presented to a tertiary referral centre at 12 weeks and 3 days gestation. Fetal USS showed megacystis, hydronephrosis of the right kidney and a multicystic left kidney. There was a one-segment thoracic hemivertebrae, and an urachal cyst. A female infant was born. Cytogenetics showed a normal female 46, XX karyotype. Microarray testing was normal on the 1Mb BAC targeted array platform (BlueGnome Cambridge). The source of DNA from F15 for sequencing was cord blood at delivery.

Fetal megacystis (HP:0010956); Abnormality of the urachus (HP:0010478); Hydronephrosis (HP:0000126); Renal cysts (HP:0000107); Hemivertebrae (HP:0002937)

F17

A 24 year old woman presented to a tertiary referral centre at 23 weeks gestation. USS revealed fetal renal agenesis and anhydramnios. The couple opted for a termination of pregnancy. Post-mortem examination showed a female fetus

appropriately grown for 23 weeks gestation. It confirmed unilateral renal agenesis accompanied by contralateral simple renal hypoplasia. Also seen were "Potter-type" facial features (low-set ears, flat nose, and forehead), bilateral talipes, discoid adrenals, and a small bladder. Cytogenetics showed a normal female 46, XX karyotype. Microarray testing was normal on the 1Mb BAC targeted array platform (BlueGnome Cambridge) and the higher resolution ISCA v2·0 60K array (Bluegnome). The source of DNA from F17 for sequencing was a tissue sample.

Edema (HP:0000969); Abnormality of the amniotic fluid (HP:0001560); Low-set ears (HP:0000369); Abnormality of adrenal morphology (HP:0011732); Hypoplasia of the bladder (HP:0005343); Renal hypoplasia (HP:0000089); Unilateral renal agenesis (HP:0000122); Potter facies (HP:0002009); Flat forehead (HP:0004425); Flat nose (HP:0000457); Hypoplasia of the thymus (HP:0000778); Excessive wrinkled skin (HP:0007392); Pulmonary hypoplasia (HP:0002089); Talipes (HP:0001883)

F18

A 26 year old woman presented to a tertiary referral centre at 21 weeks and three days gestation. Fetal USS showed an exomphalos and mild kyphosis. A live baby boy was delivered at 37 weeks and four days gestation. A small exomphalos was confirmed and cloacal exstrophy was diagnosed. In addition he had sacral dysgenesis, spina bifida, bilateral talipes, an imperforate anus, shortening of his bowel, and left-sided renal ectopia. Some of these symptoms are consistent with OEIS complex. He underwent five corrective operations within the first two years of life. His motor developmental milestones were delayed: he crawled at over one year of age and walked at 21 months. At 23 months of age he can speak over 50 words and has good understanding. Cytogenetics showed a normal male 46, XY karyotype. Microarray testing was normal on the 1Mb BAC targeted array platform (BlueGnome Cambridge) and the higher resolution ISCA v2·0 60K array (BlueGnome). The source of DNA from F18 for sequencing was cultured amniocytes.

Anal atresia (HP:0002023); Intestinal hypoplasia (HP:0005245); Omphalocele (HP:0001539); Cloacal exstrophy (HP:0010475); Ectopic kidney (HP:0000086); Motor delay (HP:0001270); Spina bifida (HP:0002414); Talipes (HP:0001883); Kyphosis (HP:0002808); Dysplastic sacrum (HP:0008455)

F19

A 26 year old woman of Indian ancestry presented to a tertiary referral centre at 20 weeks and three days gestation. On USS a fetal bilateral cleft lip and palate were visualized. A live baby boy was born. Postnatally he was also shown to have a small atrial septal defect (ASD), patent ductus arteriosis, and oesophageal atresia. He has since undergone surgery to repair the oesophagus. Karyotype was that of a normal male (46, XY). Microarray testing was normal both on the 1Mb BAC targeted array platform (BlueGnome Cambridge) and the higher resolution ISCA v2·0 array (BlueGnome). The source of DNA from F19 for sequencing was a postnatal blood sample.

Esophageal atresia (HP:0002032); Defect in the atrial septum (HP:0001631); Patent ductus arteriosus (HP:0001643); Oral cleft (HP:0000202)

F20

A 24 year old woman presented to a tertiary referral centre at 12 weeks and two days gestation. Fetal USS showed an increased nuchal translucency of 5·6mm, tricuspid regurgitation, choroid plexus cysts and an echogenic cardiac focus. The legs and feet appeared very abnormal with an extended attitude and talipes. The pregnancy subsequently miscarried and there was no post-mortem examination. Karyotype was that of a normal male (46, XY). Microarray testing was normal both on the 1Mb BAC targeted array platform (BlueGnome Cambridge) and the higher resolution ISCA v2·0 60K array (Bluegnome). The source of DNA for F20 was a chorionic villus sample.

Increased nuchal translucency (HP:0010880); Echogenic intracardiac focus (HP:0010942); Tricuspid regurgitation (HP:0005180); Choroid plexus cyst (HP:0002190); Talipes (HP:0001883); Abnormality of the lower limb (HP:0002814)

F21

A 23 year old woman presented to a tertiary referral centre at 21 weeks and six days gestation. USS showed short fetal long bones and ambiguous genitalia. A baby boy was born prematurely at 26 weeks gestation. Postnatally, he was confirmed to have ambiguous genitalia and also a cardiac ASD. He was transferred to the intensive care area of the neonatal unit and was intubated. He developed thrombocytopenia and a subsequent intraventricular haemorrhage. He died at 17 days of age secondary to lung hypoplasia and respiratory infection. The karyotype was that of a normal male (46, XY). Microarray testing was normal both on the 1Mb BAC targeted array platform (BlueGnome Cambridge) and the higher resolution ISCA v2·0 60K array (BlueGnome). The source of DNA from F21 for sequencing was cord blood at delivery.

Thrombocytopenia (HP:0001873); Premature birth (HP:0001622); Defect in the atrial septum (HP:0001631); Ambiguous genitalia (HP:0000062); Cerebral hemorrhage (HP:0001342); Pulmonary hypoplasia (HP:0002089); Respiratory tract infection (HP:0011947); Short long bones (HP:0003026)

F22

A 24 year old woman presented at 30 weeks and five days gestation to a tertiary referral centre. USS showed an isolated fetal cleft lip. A baby boy was born at 36 weeks gestation by Caesarean section for breech presentation. After birth a cleft lip and palate were confirmed and a cardiac cor triatriatum was diagnosed. He has since undergone operative procedures for both structural anomalies. The child was walking at 14 months old and had normal speech at 24 months. Karyotype was that of a normal male (46, XY). Microarray testing was normal both on the 1Mb BAC targeted array platform (BlueGnome Cambridge) and the higher resolution ISCA v2·0 60K array (BlueGnome). The source of DNA from F22 for sequencing was a tissue sample.

Abnormality of cardiac atrium (HP:0005120); Oral cleft (HP:0000202)

F23

A 31 year old woman presented at 22 weeks and three days gestation to a tertiary referral centre. USS showed findings consistent with fetal skeletal dysplasia including shortened long bones, and a “telephone receiver” appearance of the

femur and humerus. The hands and feet were difficult to visualize, the chest was small, and the ribs were shortened. An abnormality such as thanatophoric dysplasia was suggested. The couple opted for a termination of pregnancy. A subsequent post-mortem examination showed a male fetus whose measurements reflected severe osteochondrodyplasia. Skeletal examination showed disproportionate dwarfism with short limbs, a large head, and dysmorphic facial features. In addition, the chest was narrow and bell-shaped, and there was megaloencephaly. Collectively X-ray features, brain abnormalities, and histology of bones and joints were consistent with thanatophoric dysplasia type 1. The karyotype was that of a normal male (46, XY). Microarray testing was normal both on the 1Mb BAC targeted array platform (BlueGnome Cambridge) and the higher resolution ISCA v2·0 60K array (BlueGnome). The source of DNA from F23 for sequencing was a tissue sample.

Hydrops fetalis (HP:0001789); Abnormality of the liver (HP:0001392); Hypoplastic heart (HP:0001961); Low-set ears (HP:0000369); Megalencephaly (HP:0001355); Macrocephaly (HP:0000256); Hypoplastic nasal bridge (HP:0005281); Polymicrogyria (HP:0002126); Abnormality of the cerebral white matter (HP:0002500); Cortical dysplasia (HP:0002539); Pulmonary hypoplasia (HP:0002089); Bowing of the long bones (HP:0006487); Short iliac bones (HP:0100866); Bell-shaped chest (HP:0001591); Short ribs (HP:0000773); Narrow chest (HP:0000774); Platyspondyly (HP:0000926); Abnormality of the humeral epiphyseal plate (HP:0003905); Short long bones (HP:0003026); Skeletal dysplasia (HP:0002652); Disproportionate short-limb short stature (HP:0008873)

F25

A 35 old woman presented at 28 weeks and one day gestation to a tertiary referral centre. USS showed that the fetus had a right-sided hydrothorax with a mediastinal shift. A baby boy was born at term by Caesarean section weighing 3·77 kg and his length and head circumference were on the 50th centile. At 34 months of age he was meeting developmental milestones normally and was not under paediatric follow up, as he was healthy. The karyotype was that of a normal male (46, XY). Microarray testing was normal on the 1Mb BAC targeted array platform (BlueGnome Cambridge). The source of DNA from F25 for sequencing was cord blood at delivery.

Pleural effusion (HP:0002202)

F26

A 35 year old woman presented at 12 weeks and three days gestation to a tertiary referral centre. USS showed that the fetus had megacystis with possible lower urethral tract obstruction. The pregnancy miscarried and no post-mortem examination was performed. The karyotype was that of a normal male (46, XY). Microarray testing was normal both on the 1Mb BAC targeted array platform (BlueGnome Cambridge) and the higher resolution ISCA v2·0 60K array (BlueGnome). The source of DNA for F26 was a chorionic villus sample.

Increased nuchal translucency (HP:0010880); Fetal megacystis (HP:0010956); Urethral obstruction (HP:0000796)

F27 and F33

A 33 year old woman with neurofibromatosis type 2 presented at 15 weeks and six days gestation to a tertiary referral centre. USS of the fetus (F27) showed features consistent with a lower urethral tract obstruction (enlarged bladder, oligohydramnios, and echogenic parenchyma of the right kidney). The couple opted for a termination of pregnancy. Post-mortem examination showed a female fetus with appropriate growth for 16 weeks gestation. It confirmed a distended bladder secondary to urethral atresia, and also showed a recto-vesical fistula, slightly dilated ureters and kidneys, and low-set ears. The karyotype of the fetus was that of a normal female (46, XX). Microarray testing was normal both on the 1Mb BAC targeted array platform (BlueGnome Cambridge) and the higher resolution ISCA v2·0 60K array (BlueGnome). The source of DNA from F27 for sequencing was a tissue sample.

The couple went on to have a further pregnancy. An USS at 13 weeks gestation showed that the fetus (F33) had ascites and a massively dilated bladder. The pregnancy was terminated at 13 weeks gestation. A post-mortem examination showed anal and urethral atresia. The uterus was incorporated into the posterior wall of the bladder (probably pressure related), and a dilated bladder, ureters, and slightly dilated renal pelvises were visualized. F33 also had micrognathia, ambiguous genitalia, and possible hypoplasia of the cerebellar vermis. Quantitative fluorescent PCR showed no evidence of trisomy 13,18, 21, or sex chromosome aneuploidy. Multiplex ligation-dependent probe

amplification showed no evidence of deletions or duplications in the subtelomeric regions. Microarray testing was not preformed. The source of DNA from F33 for sequencing was a tissue sample.

F27: Oligohydramnios (HP:0001562); Abdominal distension (HP:0003270); Low-set ears (HP:0000369); Ambiguous genitalia (HP:0000062); Abnormality of the uterus (HP:0000130); Fetal megacystis (HP:0010956); Urethral obstruction (HP:0000796); Abnormality of the bladder (HP:0000014); Dilatation of the renal pelvis (HP:0010946); Congenital megaloureter (HP:0008676); Abnormality of the kidney (HP:0000077); Urethral atresia, female (HP:0000067); Rectal fistula (HP:0100590); Abnormality of the morphology of the rib cage (HP:0001547)

F33: Abnormality of the uterus (HP:0000130); Fetal megacystis (HP:0010956); Urethral obstruction (HP:0000796); Abnormality of the bladder (HP:0000014); Dilatation of the renal pelvis (HP:0010946); Congenital megaloureter (HP:0008676); Abnormality of the kidney (HP:0000077); Micrognathia (HP:0000347); Ambiguous genitalia (HP:0000062); Agenesis of cerebellar vermis (HP:0002335)

F28

A 28 year old woman presented at 11 weeks and six days gestation to a tertiary referral centre. USS showed that the fetus had an increased nuchal translucency of 3·5 mm, and evidence of tricuspid regurgitation. The pregnancy resulted in a live birth of a female baby. We were not able to contact the parents for follow up information. Karyotype of the fetus was that of a normal female (46, XX). Microarray testing was normal both on the 1Mb BAC targeted array platform (BlueGnome Cambridge and the higher resolution ISCA v2·0 60K array (BlueGnome). The source of DNA for F28 was a chorionic villus sample.

Increased nuchal translucency (HP:0010880); Tricuspid regurgitation (HP:0005180)

F29

A 31 year old woman presented at 12 weeks and four days gestation to a tertiary referral centre. USS showed that the fetus had an increased nuchal translucency of 5·9 mm, tricuspid regurgitation, and an intra-cardiac focus. The pregnancy resulted in a live birth of a female baby. The baby weighed 3 kg and had length and head circumference measurements between the 25th and 50th centile. At 20 months of age she was meeting developmental milestones normally. Karyotype was that of a normal female (46, XX). Microarray testing was normal both on the 1Mb BAC targeted array platform (BlueGnome Cambridge) and the higher resolution ISCA v2·0 60K array (BlueGnome). The source of DNA for F29 was a chorionic villus sample.

Increased nuchal translucency (HP:0010880); Echogenic intracardiac focus (HP:0010942); Tricuspid regurgitation (HP:0005180)

F31

A 28 year old woman presented at a tertiary referral centre at 22 weeks and four days gestation. USS showed severe fetal hydrocephalus, with a small transcerebral diameter. In addition, a complex cardiac anomaly was visualized which appeared as a probable Fallot's tetralogy variant. The stomach was not visible and there appeared to be thoracic and lumbar hemivertebrae. The couple opted to have a termination of this pregnancy, and no post-mortem examination was performed. Karyotype was that of a normal female (46, XX). Microarray testing was normal on the 1Mb BAC targeted array platform (BlueGnome Cambridge). The source of DNA for F31 was a fetal blood sample.

Hydrocephalus (HP:0000238); Tetralogy of Fallot (HP:0001636); Hemivertebrae (HP:0002937)

F32

A 37 year old woman presented at a tertiary referral centre at 20 weeks and one day gestation. USS showed that the fetus had hydrops fetalis, bilateral talipes, and fixed flexion of both wrists. The couple opted to terminate the pregnancy at 22 weeks and three days, and no post-mortem examination was performed. Karyotype was that of a

normal female (46, XX). Microarray testing was normal on the 1Mb BAC targeted array platform (BlueGnome Cambridge). The source of DNA for F32 was a fetal blood sample.

Hydrops fetalis (HP:0001789); Talipes (HP:0001883); Wrist flexion contracture (HP:0001239);

Detailed methods

Exome sequencing

All samples were whole exome sequenced at the Wellcome Trust Sanger Institute, Cambridge, UK. Genomic DNA was extracted from the samples using standard protocols, and fragmented using an ultrasonicator. 100-400 bp fragments were prepared using Illumina paired-end DNA library preparation, enriched for exonic sequences using a SureSelect_All_Exon_50Mb_GRCh37_hs37d5 and then sequenced using the HiSeq™ platform (Illumina) as paired-end 75-bp reads according to the manufacturer's protocol. All samples were indexed and pooled four to a lane for sequencing, except P5, which was not pooled. In all other respects this sample was treated the same as the others, but it does have higher coverage (see Figure S1).

Reads were mapped to reference GRCh37_hs37d5. Picard MarkDuplicates (http://picard.sourceforge.net) was used to mark molecular duplicates in the BAM files. GATK was then used to realign reads near potential indel sites within the target variant calling regions (bait regions +/- 100bp). (2) Finally GATK was used to recalibrate bases quality scores. From these improved BAM files, variants were called using three different callers. First, variants are called using SAMtools mpileup options -d 500 -C50 -m3 -F0·002 and default settings, and filtered using vcfutils.pl utility and options -p -d 4 -D 1200 and the default settings. (3) Second, GATK was used to call indels using IndelGenotyper, and single nucleotide variants using GATK UnifiedGenotyper. Poor quality sites were filtered out near indels and using hard filters. Third, indels were called using Dindel. (4) In each case, variants are only reported within the bait regions +/- 100bp (an extra 10bp is allowed for Dindel calling). In each case, the following annotation was added to the VCF files; gene name, consequence, Polyphen score, and SIFT score from the ensembl v64 genebuild using the Ensembl Variant Effect Predictor v2·2, allele frequency information from 1000 Genomes Project (20101123 sequence release), and identifiers from Database of Single Nucleotide Polymorphisms (dbSNP) build ID: 134. (5-8)

Identification of *de novo* SNPs and indels

To identify *de novo* mutations we used *De Novo* Gear pipeline version 0·6·2. (9, 10) We had a two-tier filtering strategy to prioritize the variants called by *De Novo* Gear. For genes not known to cause developmental disease (identified using the Developmental Disorder Gene2Phenotype (DDG2P) gene list available at decipher.sanger.ac.uk) we filtered out variants with minor allele frequency >0·01, in non-coding regions, depth <10x (in any of trio), in a

tandem repeat or segmental duplication, we removed variants which occur in >10% of either parental read, and those where the calls in the VCF files were not consistent with a *de novo* mode of inheritance. Finally we visually inspected plots of the reads using the Integrative Genomics Viewer (IGV) and removed variants that appeared incorrectly mapped. (11) For genes in DDG2P we used a slightly less stringent filtering process. We removed variants with minor allele frequency >0·01, in non-coding regions, and those that appeared incorrectly mapped on IGV plots.

To calculate whether our final list of *de novo* mutations is enriched for functional mutations over what would be expected by chance, we calculated that the proportion of *de novo* variants in exons expected to be functional, by chance is 71·4%. (12) We compared this to the proportion of *de novo* variants that are functional in our cohort using a binomial test. To calculate the probability that a given number of functional *de novo* variants will occur in the same gene in this cohort by chance, we calculated the number that are expected to occur using the known exome mutation rate, and the proportion of variants that are expected to be functional, taking into account the length of the coding sequence of the gene of interest. (12, 13) We compared this to the observed number of such variants.

Identification of inherited recessive and X-linked SNPs and indels

For each of the samples, we merged the VCF files from the different variant callers using VCFtools. (14) We identified inherited SNPs and indels under different Mendelian models using in-house Python scripts. We only considered variants that passed the callers' quality filters, were functional (predicted protein consequences were essential splice site, stop gained, frameshift coding, non synonymous, or stop lost), and had an allele frequency of <0·01 in both the 1000 Genomes project, and an internal control cohort of 2172 individuals exome sequenced at the same laboratory, using the same pipelines and analysis methods. We also only considered variants in which the genotypes of the three members of the trio were consistent with inherited recessive (homozygous or compound heterozygous) or X-linked model of inheritance, with unaffected parents.

Validation of SNVs and indels using Sanger sequencing

We whole genome amplified ~50 ng genomic DNA from each sample using Illustra Genomiphi V3 ready-to-go kit (GE Healthcare Life Sciences, Buckinghamshire, UK) according to the manufacturer's instructions. We used this as a template to amplify a fragment containing each the variant of interest in the relevant trios using REDTaq® DNA

Polymerase (Sigma-Aldrich, Dorset, UK) and capillary sequenced using BigDye v31 kit and ABI 3730 sequencer according to the manufacturers' instructions.

Identification of CNVs from exome data

To call CNVs from the exome data we used CoNVex (manuscript in preparation, ftp://ftp.sanger.ac.uk/pub/users/pv1/CoNVex/). Not to be confused with ADTEx, previously known also as CoNVex. (15) CoNVex detects copy number variation from exome data using comparative read depth. It corrects for technical variation between samples and detects copy number variable segments using a heuristic error-weighted score and the Smith-Waterman algorithm. It detects deletions and duplications of targeted sequences from few hundred base pairs in size to a few Mb or more.

We called a total of 12,909 CNVs across the 86 samples. To identify candidate CNVs we filtered this initial set as follows: we only kept CoNVex confidence score >=10, overlap within known common CNVs < 0·5, internal frequency of CNV in the dataset <5%, contains >0 protein-coding genes, covered by >1 probe, removed excessively noisy samples. We identified putative *de novo* and inherited X-linked CNVs in the fetuses, and inspected plots of regional $\log_2$ ratios in the family members and filtered out likely technical artifacts.

Supplementary Figures

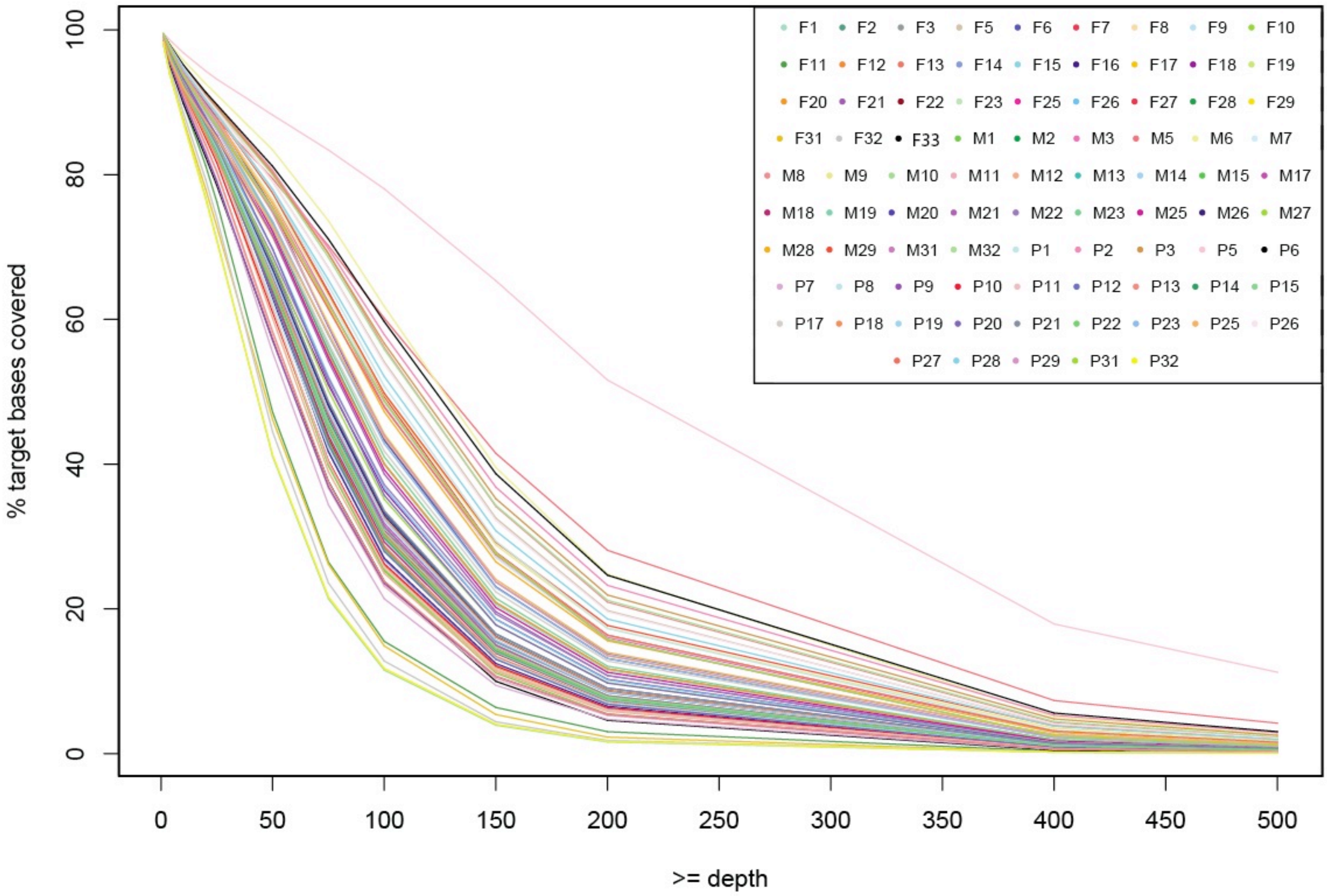

Figure S1: Target coverage of exome sequencing reads by sample. P5 has higher coverage, as it was not sequenced as part of a pool.

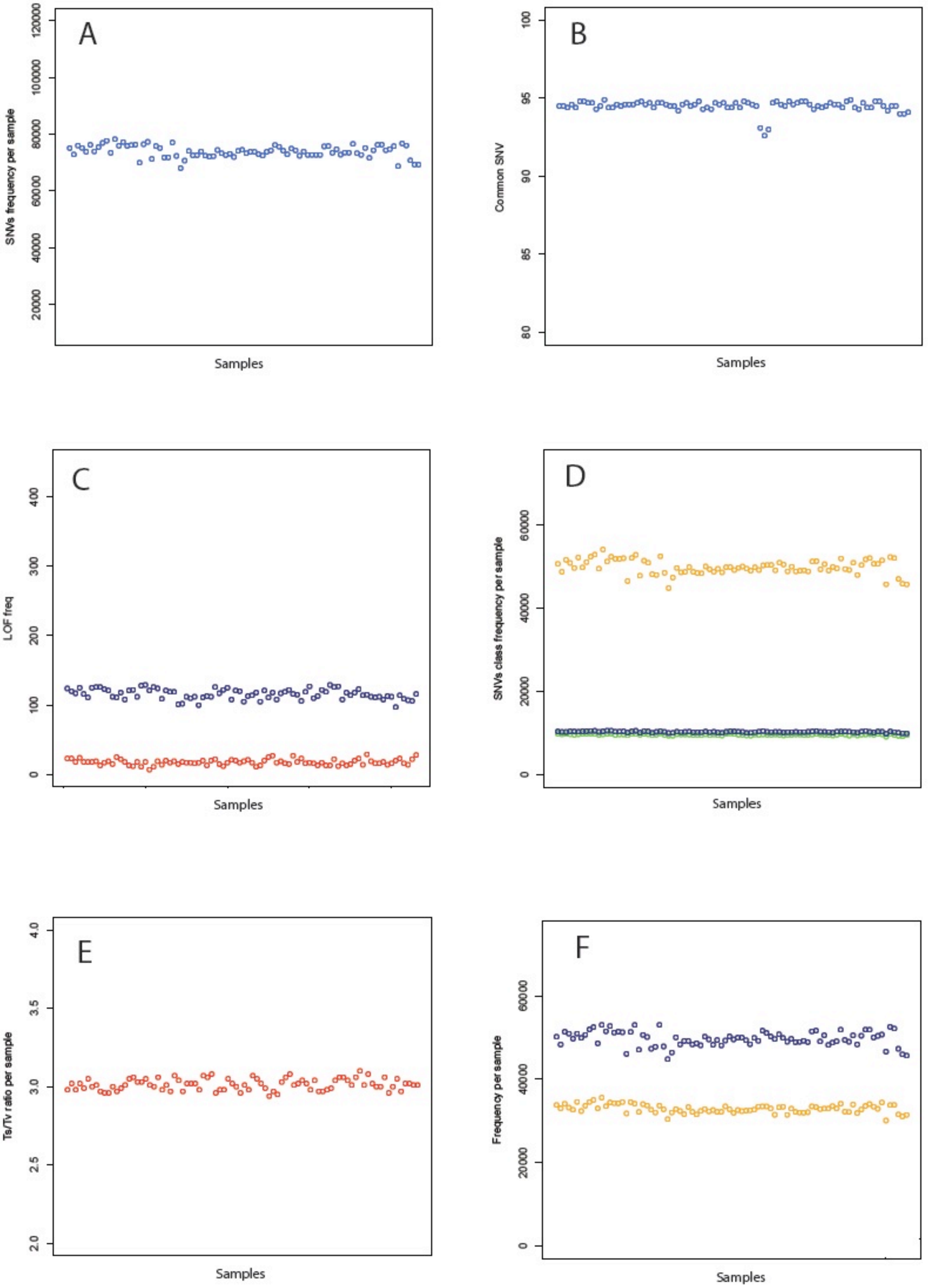
A
SNVs frequency per sample
20000
40000
60000
80000
100000
120000
Samples
B
Common SNV
80
85
90
95
100
Samples
C
LOF freq
0
100
200
300
400
Samples
D
SNVs class frequency per sample
0
20000
40000
60000
Samples
E
Ts/Tv ratio per sample
2.0
2.5
3.0
3.5
4.0
Samples
F
Frequency per sample
0
20000
40000
60000
Samples

Figure S2: Quality control metrics of SNVs from merged variant call files. For each plot, the 86 samples are shown along the x axis in the following order: F1, M1, P1, F2, M2, P2, F3, M3, P3, F5, M5, P5, F6, M6, P6, F7, M7, P7, F8, M8, P8, F9, M9, P9, F10, M10, P10, F11, M11, P11, F12, M12, P12, F13, M13, P13, F14, M14, P14, F15, M15, P15, F16, F17, M17, P17, F18, M18, P18, F19, M19, P19, F20, M20, P20, F21, M21, P21, F22, M22, P22, F23, M23, P23, F25, M25, P25, F26, M26, P26, F27, M27, P27, F33, F28, M28, P28, F29, M29, P29, F31, M31, P31, F32, M32, P32. **(A)** Number of high-quality SNVs per sample. **(B)** Percent of SNVs that are common (≥1% population frequency) per sample. The cluster of three samples with a lower percentage of common SNVs represents F19, M19 and P19. These individuals are of Indian ancestry, whereas most of the cohort is of European ancestry. **(C)** Number of loss of function SNVs per sample. Common (≥1%) are shown in blue and rare (<1%) are shown in red. **(D)** Number of SNVs per sample that are functional (green), silent (blue) and other (yellow). **(E)** Transition/transversion ratio per sample. **(F)** Number of SNVs per sample that are heterozygous (blue), and homozygous (yellow).

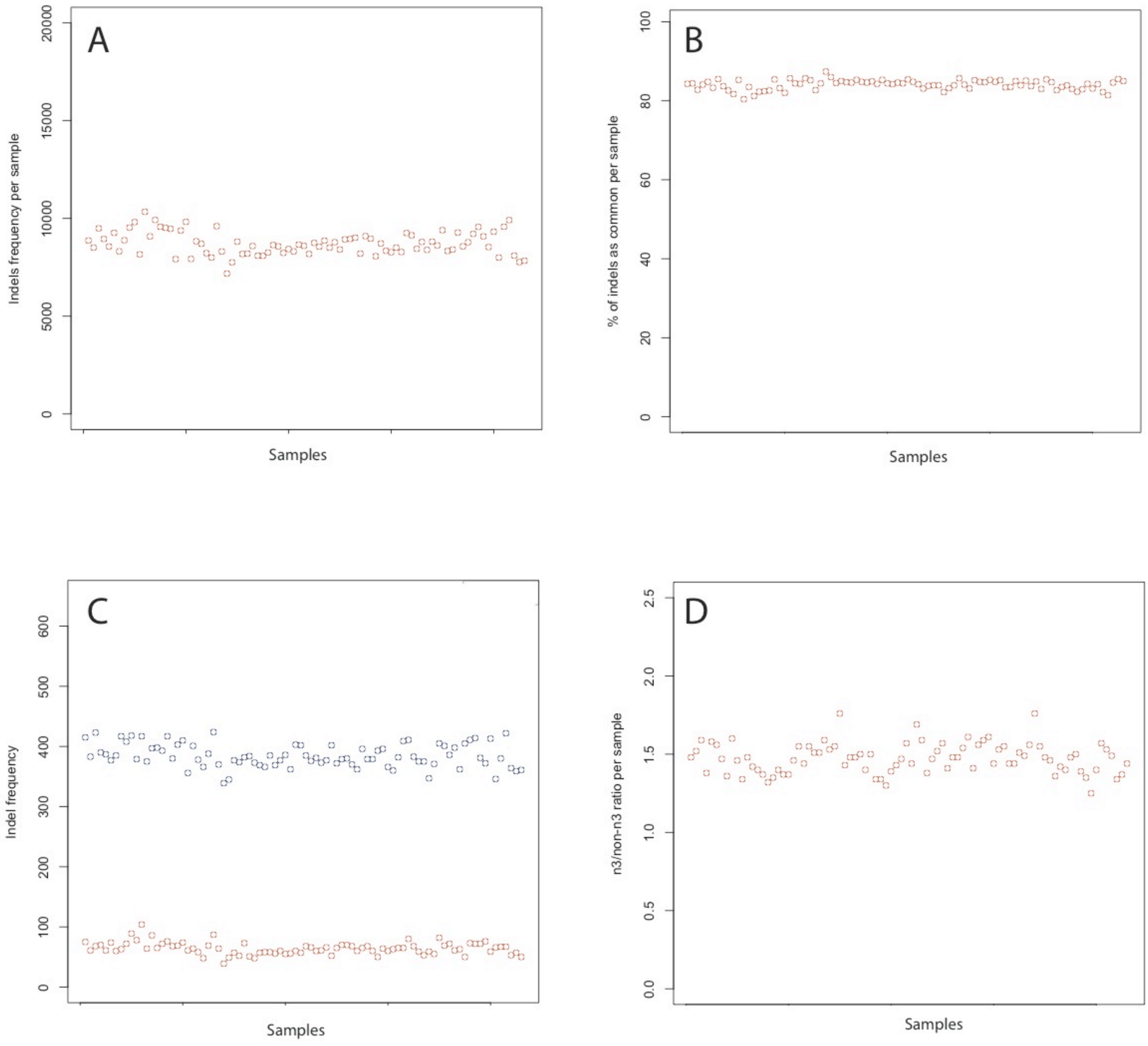

Figure S3: Quality control metrics of INDELs from merged variant call files. For each plot, the 86 samples are shown along the x axis in the same order as in Figure S2. **(A)** Number of high-quality indels per sample. **(B)** Percent of indels that are common (≥1% population frequency) per sample. **(C)** Number of coding indels per sample. Common (≥1%) are shown in blue and rare (<1%) are shown in red. **(D)** Ratio of coding indels with length that is a multiple of three against coding indels with length that is not a multiple of three, per sample.

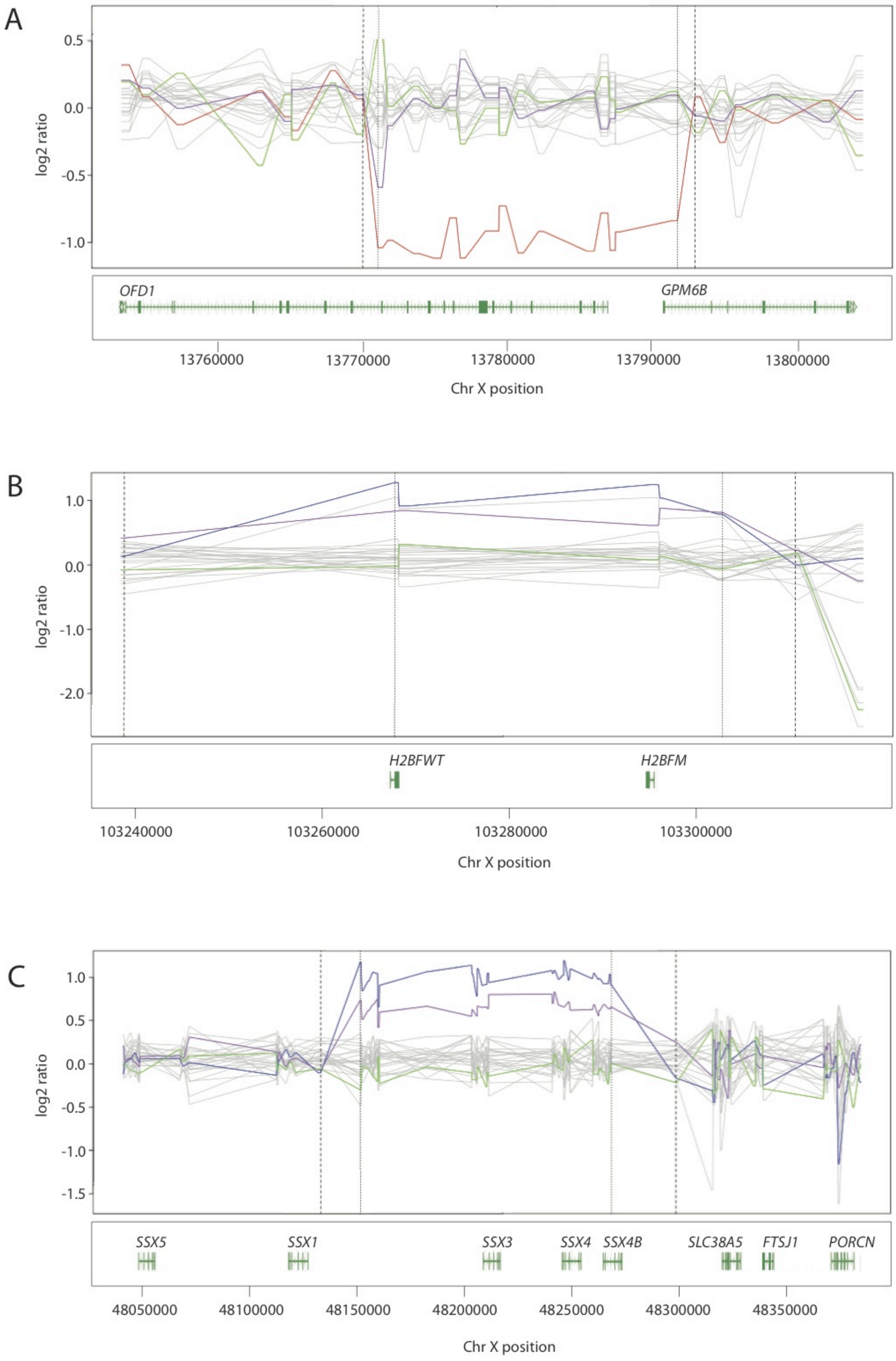
A
0.5
0.0
-0.5
-1.0
log2 ratio
OFD1
GPM6B
13760000
13770000
13780000
13790000
13800000
Chr X position
B
1.0
0.0
-1.0
-2.0
log2 ratio
H2BFWT
H2BFM
103240000
103260000
103280000
103300000
Chr X position
C
1.0
0.5
0.0
-0.5
-1.0
-1.5
log2 ratio
SSX5
SSX1
SSX3
SSX4
SSX4B
SLC38A5
FTSJ1
PORCN
48050000
48100000
48150000
48200000
48250000
48300000
48350000
Chr X position

Figure S4: Three rare, high-confidence CNVs that contain protein coding exons were identified from the exome sequencing data using CoNVex. In each plot the x axis indicates the genomic coordinates. The top panel indicates the normalised $\log_2$ ratio of the exome read depth, compared to a group of controls. The red line shows the fetuses data, where the variant is a deletion, and the blue line shows the fetuses data where the variant is a duplication. The purple line shows the mother's data and the green line the father's data. The grey lines show the data of control samples. The vertical small dashed lines show the minimum deleted/duplicated region and the vertical wide dashed lines show the maximum deleted/duplicated region. The bottom panel shows the protein-coding genes present in the region. **(A)** This shows a *de novo* deletion in chromosome X of F14 (female) that encompasses part of *OFD1*. The deleted region covers 15 probe regions and has a CoNVex score of 26. This deletion is highly likely to be causal of the phenotype. **(B)** This shows a duplication in chromosome X in F3 (male) that was inherited from the mother. This duplication covers 5 probe regions and has a CoNVex score of 17. **(C)** This shows a duplication in chromosome X in F19 (male) that was inherited from the mother. This duplication covers 32 probe regions and has a CoNVex score of 68.

Supplementary Tables

ID	Gb	Number reads mapped	Number HMQ reads mapped to PTR	% Q20 bases	>=1x bp %) PTR	>=10x bp %) PTR	Mean depth PTR	Number coding variants
F1	9·13	105237798	71288090	95·71	99·25	94·11	106·274	21826
F2	9·28	105651453	71507176	95·75	99·03	93·53	106·564	21667
F3	9·40	111087061	76307901	95·68	98·95	93·11	114·432	21742
F5	12·90	143381090	92699881	95·66	99·42	95·02	137·72	21954
F6	9·52	113758366	75797156	95·83	99·16	94·3	113·295	21940
F7	10·98	129332290	84423053	95·6	99·18	94·36	125·512	21552
F8	10·71	126963426	84367449	95·64	99·37	94·78	125·866	21687
F9	10·64	124319978	83754651	95·7	99·25	94·49	125·248	21742
F10	6·47	78578441	53387862	95·8	98·78	92·15	79·831	21440
F11	4·86	59892938	40775602	95·85	98·62	89·9	61·05	20857
F12	6·59	78886566	53976303	95·75	98·75	91·81	80·52	21367
F13	7·01	84459517	57086795	95·82	98·95	92·23	85·211	21237
F14	6·94	84250165	55239595	95·76	98·98	92·82	82·435	21663
F15	7·25	89037614	58512496	95·76	98·78	92·05	87·287	21155
F16	6·89	83524378	55517406	95·68	99·2	93·09	83·102	21956
F17	6·89	83702346	56395887	95·77	98·82	92·42	84·406	21640
F18	8·80	98067006	66147741	96·59	98·62	91·17	98·053	20964
F19	7·32	86890454	58908353	95·53	98·92	92·07	87·821	21779
F20	9·11	106021109	70831428	96·63	98·95	92·37	105·403	21281
F21	8·85	105145649	68895929	96·67	99·07	92·73	102·558	21127
F22	7·24	83988636	56904719	95·5	99·06	92·78	84·907	21498
F23	7·30	84897815	58600063	95·45	98·82	91·95	87·365	21353
F25	7·52	89947422	59597856	95·49	98·95	92·04	88·807	21513
F26	8·51	99076689	66648868	96·6	98·84	92·11	99·192	20982
F27	7·37	87886539	59205640	95·53	98·84	92·44	88·366	21535
F28	7·76	92688509	62500308	95·43	98·85	92·54	93·196	21525
F29	9·76	113256975	76111740	96·6	98·93	92·97	113·526	21219
F31	6·34	64334697	38825777	96·56	98·17	87·64	57·866	20468
F32	6·28	63976357	37322925	96·44	98·46	88·61	55·537	21046
F33	8·16	83931131	49286255	96·58	98·64	89·88	73·419	21075
M1	7·04	78462478	52645663	95·44	98·84	92·15	78·481	21498
M2	7·60	88704517	59986920	95·54	98·81	92·15	89·333	21499
M3	10·90	120910976	82707758	96·16	98·99	93·98	123·515	21784
M5	13·69	158875993	110411666	95·87	98·73	93·19	165·606	21456

M6	14·14	154001304	104330917	95·66	99·36	95·82	155·316	22028
M7	11·07	125734689	82706691	96·1	99·16	94·58	123·255	21622
M8	12·53	143667239	95419993	96·03	99·17	94·99	142·143	21817
M9	10·95	125313300	85590849	96·18	98·94	93·72	127·864	21612
M10	12·70	142999829	95663391	96·13	99·16	94·78	142·556	21956
M11	6·13	73463853	50195030	96·2	98·48	90·88	75·003	20901
M12	6·55	76961980	52632594	96·17	98·67	91·75	78·669	21451
M13	7·32	88045919	60213492	96·13	98·51	91·41	90·143	21258
M14	7·45	88073298	58095399	96·11	98·89	92·49	86·586	21152
M15	7·05	85060165	56736873	96·19	98·85	92·43	84·692	20934
M17	7·63	90190994	60229898	96·09	98·86	92·76	89·792	21449
M18	7·18	86089729	57601439	96·18	98·74	91·84	85·68	20930
M19	7·40	86933766	58492263	96·15	98·86	92·63	87·242	22220
M20	7·90	93022110	62693258	95·86	98·72	92·12	93·795	21422
M21	7·76	89702184	60860845	95·9	98·67	91·94	90·91	21212
M22	8·46	99184196	67534892	95·84	98·64	91·98	100·657	21408
M23	9·34	110248844	72503603	95·8	99·05	93·61	107·912	21670
M25	8·81	104859853	69963332	95·77	98·97	93·42	104·385	21374
M26	8·02	90982195	62052636	95·85	98·74	92·31	92·442	21378
M27	8·41	94953031	65123188	95·9	98·72	92·33	97·14	21177
M28	10·54	120218757	81636876	97·13	99·1	93·95	121·798	21812
M29	11·16	127031875	86596684	97·08	99·14	94·3	130·433	21597
M31	14·62	147870416	81006172	96·57	99·34	93·57	120·641	21736
M32	5·68	58636104	35173157	96·5	98·15	87·36	52·393	20791
P1	11·97	128112177	85699745	96·98	99·32	94·54	128·031	21561
P2	13·10	151199563	100554052	97·12	99·39	94·93	150·412	21516
P3	12·75	147616139	97431748	97·2	99·42	95·07	145·569	21968
P5	25·34	260922095	174856038	96·96	99·58	96·84	260·148	21617
P6	14·15	154051325	103897082	95·79	99·37	95·17	154·839	21319
P7	5·87	71324318	48099786	97·81	98·73	90·69	71·891	21121
P8	6·92	83365682	55948619	97·83	98·84	91·48	83·722	21189
P9	6·25	76299997	50521398	97·75	98·73	90·39	75·181	21042
P10	6·64	81504471	54187949	97·78	98·94	91·66	80·854	21339
P11	6·80	82578652	53758221	97·73	99·1	92·28	80·136	21611
P12	6·95	85793300	56321179	97·73	99·05	92·25	83·975	21454
P13	6·31	75218043	51049757	97·78	98·8	91·15	76·128	21229
P14	7·13	87194222	58646676	97·8	98·98	92·27	87·73	21445
P15	7·41	88276180	59527162	97·43	99·01	92·44	88·824	21636
P17	9·19	110105714	73688831	97·47	99·18	93·41	110·281	21268
P18	7·78	93791429	61532376	97·39	98·95	91·71	91·506	21431

P19	7·84	95133699	61501500	97·39	99·11	92·53	91·594	21757
P20	8·52	101306936	65921431	97·39	99·19	93·29	98·197	21340
P21	7·79	94992474	62992323	97·4	99·04	92·52	94·03	21352
P22	7·41	87344469	58820342	97·44	98·96	92·12	87·688	21274
P23	9·87	113611202	75143669	96·28	99·22	93·66	111·818	21781
P25	9·41	113945480	76510093	96·69	98·76	92·09	114·498	21325
P26	11·95	138130284	92137474	96·27	99·36	94·84	137·371	21831
P27	10·68	124740809	82888871	96·22	99·22	93·7	123·255	21487
P28	11·81	136620478	89608716	96·23	99·36	94·64	133·306	21526
P29	9·89	116682042	76685316	96·29	99·32	94·22	114·252	21583
P31	14·66	149006530	81272187	96·53	99·5	93·76	120·872	21304
P32	5·74	59099655	35398578	96·59	98·31	87·25	52·7	20983

Table S1: Exome sequencing coverage and quality metrics. Mapped to GRCh37_hs37d5 (Human). Gb = gigabases sequenced. Q20 = Number of bases with a phred-like calibrated quality score of 20 or above (raw archive data). A base with a quality score of 20 has a probability of being an incorrect call of one in 100 (so 99% will be correct). HMQ = high mapping quality (>Q30), PTR = primary target region. Number coding variants = number of high-quality coding variants in each merged VCF file.

Fetus ID	CHR	POS	REF	ALT	GN	CQ	PolyPhen	SIFT	HI	GERP	N_ REF	N_ ALT	P
F2	16	9857047	G	A	*GRIN2A*	NS	probably_damaging(1)	deleterious(0)	0.307	4.68	29	24	0.29
F3	11	7618837	G	C	*PPFIBP2*	NS	probably_damaging(1)	tolerated(0.12)	0.105	4.83	28	16	0.048
F6	11	33677654	C	T	*C11orf41*	STOP	.	.	0.141	-0.417	43	46	0.66
F6	12	56567575	G	A	*SMARCC2*	STOP	.	.	0.995	3.77	122	102	0.1
F6	17	29562669	G	A	*NF1*	NS	possibly_damaging(0.501)	deleterious(0.02)	0.526	4.57	146	133	0.24
F6	20	39813788	G	A	*ZHX3*	S	.	.	0.134	-2.91	9	4	0.13
F7	2	210694087	G	A	*UNC80*	NS	possibly_damaging(0.444)	tolerated(0.33)	.	4.65	138	136	0.48
F7	20	44190748	C	T	*WFDC8*	SPLICE	.	.	0.057	3.18	28	30	0.65
F8	1	160811672	G	T	*CD244*	NS	unknown(0)	tolerated(1)	0.029	-6.43	33	38	0.76
F9	2	205829965	G	C	*PARD3B*	NS	probably_damaging(0.954)	tolerated(0.39)	0.456	4.59	79	25	5.3×10^{-8}
F10	8	20069263	G	T	*ATP6V1B2*	NS	probably_damaging(1)	deleterious(0)	0.835	4.62	26	20	0.23
F10	9	91994007	C	T	*SEMA4D*	NS	benign(0)	deleterious(0)	0.19	3.15	10	7	0.31
F14	1	28099859	C	T	*STX12*	NS	benign(0.086)	tolerated(0.81)	0.525	3.66	8	12	0.87
F14	4	44450177	C	T	*KCTD8*	NS	possibly_damaging(0.597)	deleterious(0.04)	0.376	4.76	14	13	0.5
F15	10	128830000	G	A	*DOCK1*	NS	benign(0.005)	tolerated(0.29)	0.096	4.87	147	158	0.75
F16	11	7618837	G	C	*PPFIBP2*	NS	probably_damaging(1)	tolerated(0.12)	0.105	4.83	18	19	0.63
F18	3	58639419	G	A	*FAM3D*	NS	probably_damaging(1)	deleterious(0.04)	0.048	3.95	65	44	0.027
F18	12	123444538	G	A	*ABCB9*	NS	probably_damaging(0.999)	tolerated(0.16)	0.237	4.27	7	8	0.7
F19	2	205983695	G	A	*PARD3B*	NS	benign(0.084)	tolerated(0.96)	0.456	0.156	67	56	0.18
F19	3	132230069	T	C	*DNAJC13*	S	.	.	0.34	3.24	45	37	0.22
F19	17	5461819	G	C	*NLRP1*	NS	benign(0.01)	tolerated(0.67)	0.055	-3.09	30	31	0.6
F20	12	48369853	C	A	*COL2A1*	NS	unknown(0)	deleterious(0)	0.96	4.57	22	30	0.89
F22	10	71175853	G	A	*TACR2*	NS	probably_damaging(1)	deleterious(0.02)	0.113	4.86	11	16	0.88
F23	4	1806099	A	G	*FGFR3*	NS	possibly_damag	deleterious(0	0.834	2.57	57	42	0.08

							ing(0.753)	)					
F25	3	47727627	G	A	*SMARCC1*	STOP	.	.	0.86	3.48	17	15	0.43
F25	10	118359676	C	T	*PNLIPRP1*	NS	probably_damaging(0.982)	deleterious(0.03)	0.272	2.54	77	57	0.05
F26	1	202722193	C	A	*KDM5B*	NS	probably_damaging(1)	deleterious(0)	.	4.74	45	24	0.0077
F26	8	74334894	T	G	*STAU2*	NS	probably_damaging(0.957)	deleterious(0.02)	0.477	4.43	48	37	0.14
F27	2	106687405	A	G	*C2orf40*	NS	unknown(0)	.	0.142	-0.254	20	14	0.2
F27	11	15260600	G	A	*INSC*	NS	benign(0)	tolerated(0.63)	0.205	1.39	10	12	0.74
F28	19	55748185	T	C	*PPP6R1*	NS	benign(0.319)	tolerated(0.33)	.	1.89	27	29	0.66
F31	12	50047598	G	C	*FMNL3*	NS	possibly_damaging(0.85)	deleterious(0.02)	0.103	-4.16	38	24	0.049
F33	10	102249809	C	A	*SEC31B*	NS	benign(0.017)	tolerated(0.67)	0.058	2.08	21	5	0.0012
F33	X	13645272	G	A	*EGFL6*	S	.	.	0.086	1.59	111	92	0.1

Table S2: *de novo* SNPs in cohort of fetuses with structural abnormalities. These have all been validated using capillary sequencing. None were in databases of known SNPs except F6 chr17:29562669 which has ID CM000800 in HGMD and F23 chr4:1806099 which has ID CM960657 in HGMD or COSM718 in COSMIC. NS = non-synonymous coding variant; S = synonymous coding variant STOP= stop codon gained; SPLICE = essential splice site; CQ = consequence of mutation; HI = haploinsufficiency score; N_ REF = number of sequencing reads that support the reference allele; N_ ALT = number of sequencing reads that support the alternate allele; P = p value from binomial test to test whether the proportion of sequencing reads that support the alternate allele is significantly less than 0.5 (Bonferroni-corrected threshold of significance = 0.00147).

ID	GDR	GT	CHR	POS	REF	ALT	VARIANT_ID	Gene	CQ	PolyPhen	SIFT
F1	M	Hemi	X	129146962	C	T	rs141901231	*BCORL1*	NS	unknown(0)	deleterious(0.02)
F1	M	Hemi	X	34149726	G	A	.	*FAM47A*	NS	probably_damaging(0.994)	deleterious(0.01)
F1	M	Comp het	11	93806297	C	G	.	*HEPHL1*	NS	benign(0.025)	tolerated(0.25)
F1	M	Comp het	11	93808384	A	G	.	*HEPHL1*	NS	possibly_damaging(0.944)	deleterious(0.03)
F1	M	Hemi	X	108868195	C	A	.	*KCNE1L*	STOP	0	0
F1	M	Hemi	X	151869653	A	G	rs150557342	*MAGEA6*	NS	benign(0)	tolerated(1)
F1	M	Comp het	8	48719844	G	A	rs8178216	*PRKDC*	NS	benign(0)	tolerated(0.88)
F1	M	Comp het	8	48848319	C	A	.	*PRKDC*	NS	possibly_damaging(0.943)	tolerated(0.18)
F1	M	Hemi	X	117960384	G	A	.	*ZCCHC12*	NS	benign(0.079)	tolerated(0.19)
F1	M	Comp het	19	12358606	G	A	.	*ZNF44*	NS	benign(0.389)	deleterious(0.04)
F1	M	Comp het	19	12383893	G	A	.	*ZNF44*	NS	benign(0.018)	tolerated(1)
F2	F	Comp het	19	49113161	C	T	.	*FAM83E*	NS	probably_damaging(0.998)	tolerated(0.41)
F2	F	Comp het	19	49113215	G	A	.	*FAM83E*	NS	probably_damaging(1)	deleterious(0)
F2	F	Comp het	4	37445867	C	T	.	*KIAA1239*	NS	probably_damaging(0.966)	deleterious(0.04)
F2	F	Comp het	4	37446545	C	A	.	*KIAA1239*	NS	probably_damaging(0.998)	tolerated(0.57)
F2	F	Comp het	20	36868106	G	A	rs11699859	*KIAA1755*	NS	benign(0.186)	tolerated(0.51)
F2	F	Comp het	20	36870301	C	T	.	*KIAA1755*	NS	possibly_damaging(0.671)	deleterious(0.01)
F2	F	Comp het	20	60886088	C	T	.	*LAMA5*	NS	benign(0.012)	tolerated(0.18)
F2	F	Comp het	20	60892813	G	A	rs140777270	*LAMA5*	NS	probably_damaging(0.982)	deleterious(0.01)
F2	F	Comp het	1	222802423	G	A	.	*MIA3*	NS	possibly_damaging(0.664)	deleterious(0.02)
F2	F	Comp het	1	222802652	T	C	.	*MIA3*	NS	benign(0)	tolerated(0.39)
F3	M	Comp het	16	1470583	C	G	rs45490596	*C16orf91*	NS	probably_damaging(0.997)	deleterious(0.01)

F3	M	Comp het	16	1476330	T	C	rs72779224	*C16orf91*	NS	unknown(0)	0
F3	M	Comp het	9	90500202	A	G	.	*C9orf79*	NS	probably_damaging(0.962)	tolerated(0.19)
F3	M	Comp het	9	90502542	T	C	.	*C9orf79*	NS	possibly_damaging(0.605)	tolerated(0.08)
F3	M	Comp het	17	20799179	C	G	.	*CCDC144NL*	NS	possibly_damaging(0.484)	deleterious(0)
F3	M	Comp het	17	20799281	G	A	rs141096337	*CCDC144NL*	NS	probably_damaging(0.996)	tolerated(0.17)
F3	M	Hemi	X	49104709	C	T	rs143790434	*CCDC22*	NS	probably_damaging(0.999)	deleterious(0.01)
F3	M	Comp het	6	138752868	C	A	.	*NHSL1*	NS	benign(0.002)	tolerated(1)
F3	M	Comp het	6	138794490	G	A	.	*NHSL1*	NS	benign(0)	tolerated(0.5)
F3	M	Hemi	X	9863050	C	T	.	*SHROOM2*	NS	probably_damaging(0.994)	deleterious(0.03)
F5	M	Comp het	8	12957657	C	T	rs140340878	*DLC1*	NS	benign(0.046)	tolerated(0.58)
F5	M	Comp het	8	13356860	G	C	rs61757614	*DLC1*	NS	probably_damaging(0.998)	tolerated(0.36)
F5	M	Hemi	X	119394834	G	A	.	*FAM70A*	NS	benign(0.395)	tolerated(0.13)
F5	M	Hemi	X	31089928	G	A	rs140427727	*FTHL17*	NS	probably_damaging(1)	deleterious(0)
F5	M	Hemi	X	135430934	C	A	rs141778568	*GPR112*	NS	probably_damaging(0.995)	tolerated(0.38)
F5	M	Hemi	X	99551442	G	C	.	*PCDH19*	NS	benign(0)	tolerated(1)
F5	M	Hemi	X	114426292	G	A	.	*RBMXL3*	NS	probably_damaging(0.994)	deleterious(0)
F5	M	Comp het	2	179430460	A	G	.	*TTN*	NS	benign(0)	0
F5	M	Comp het	2	179497758	A	G	.	*TTN*	NS	benign(0.014)	0
F5	M	Comp het	2	179579172	C	T	.	*TTN*	NS	unknown(0)	0
F5	M	Hemi	X	117528073	A	C	.	*WDR44*	NS	benign(0.002)	tolerated(0.23)
F6	F	Hom	19	41754430	G	A	rs35538872	*AXL*	NS	probably_damaging(1)	tolerated(0.06)
F6	F	Comp het	7	30818142	T	G	.	*FAM188B*	NS	probably_damaging(1)	deleterious(0)
F6	F	Comp het	7	30825544	C	A	rs75843887	*FAM188B*	NS	benign(0.038)	tolerated(0.06)
F6	F	Comp	7	103141235	G	A	.	*RELN*	NS	benign(0)	tolerated(0.1

		het									6)
F6	F	Comp het	7	103205827	G	C	rs2229860	*RELN*	NS	probably_damaging(1)	deleterious(0.03)
F6	F	Comp het	1	8418331	C	T	.	*RERE*	NS	probably_damaging(0.998)	deleterious(0)
F6	F	Comp het	1	8418909	C	T	rs138814161	*RERE*	NS	probably_damaging(0.997)	deleterious(0.03)
F7	F	Comp het	19	9018166	A	G	rs77435151	*MUC16*	NS	benign(0.094)	0
F7	F	Comp het	19	9082960	G	A	.	*MUC16*	NS	unknown(0)	0
F7	F	Comp het	13	45148705	TTGC	T	rs76851453	*TSC22D1*	NS	0	0
F7	F	Comp het	13	45149973	G	A	rs140514784	*TSC22D1*	NS	possibly_damaging(0.893)	tolerated(0.12)
F7	F	Comp het	2	179399071	G	A	rs140319117	*TTN*	NS	probably_damaging(1)	0
F7	F	Comp het	2	179431633	C	T	rs72648206	*TTN*	NS	probably_damaging(0.989)	0
F7	F	Comp het	2	179641112	C	A	rs141213991	*TTN*	NS	benign(0.29)	0
F8	M	Comp het	2	160688217	T	C	rs113023766	*LY75-CD302*	NS	benign(0.011)	tolerated(0.25)
F8	M	Comp het	2	160738803	G	A	rs35675007	*LY75-CD302*	NS	probably_damaging(0.982)	tolerated(0.19)
F8	M	Hemi	X	153040414	C	T	.	*PLXNB3*	STOP	0	0
F8	M	Hemi	X	16870553	C	T	.	*RBBP7*	NS	benign(0.001)	tolerated(0.28)
F8	M	Hemi	X	99920314	G	T	.	*SRPX2*	NS	possibly_damaging(0.605)	deleterious(0.01)
F8	M	Comp het	2	179399677	C	T	rs34924609	*TTN*	NS	benign(0.361)	0
F8	M	Comp het	2	179412829	C	T	rs72648251	*TTN*	NS	possibly_damaging(0.449)	0
F8	M	Comp het	2	179591953	C	G	.	*TTN*	NS	unknown(0)	0
F8	M	Comp het	16	74937918	C	T	.	*WDR59*	NS	benign(0.154)	tolerated(0.55)
F8	M	Comp het	16	74990380	G	A	.	*WDR59*	NS	possibly_damaging(0.588)	tolerated(0.18)
F9	M	Comp het	7	48318614	T	G	.	*ABCA13*	NS	possibly_damaging(0.808)	0
F9	M	Comp het	7	48547481	C	T	rs76060602	*ABCA13*	NS	probably_damaging(0.999)	0
F9	M	Hemi	X	152814163	G	A	.	*ATP2B3*	NS	benign(0.001)	tolerated(0.06)

F9	M	Hemi	X	49103316	G	A	.	*CCDC22*	NS	probably_damaging(0.99)	tolerated(0.28)
F9	M	Comp het	3	130300740	C	T	.	*COL6A6*	STOP	0	0
F9	M	Comp het	3	130381038	G	A	.	*COL6A6*	NS	benign(0.084)	0
F9	M	Comp het	20	57428948	G	C	rs61749696	*GNAS*	NS	unknown(0)	tolerated(0.4)
F9	M	Comp het	20	57430118	C	G	rs74897360	*GNAS*	NS	benign(0.011)	tolerated(0.46)
F9	M	Comp het	10	30315676	G	A	rs61732650	*KIAA1462*	NS	benign(0)	tolerated(1)
F9	M	Comp het	10	30316500	A	ACTG	.	*KIAA1462*	NS	0	0
F9	M	Comp het	7	100679024	A	G	rs151245477	*MUC17*	NS	unknown(0)	0
F9	M	Comp het	7	100685477	A	T	.	*MUC17*	NS	probably_damaging(0.999)	0
F9	M	Comp het	16	2806466	C	T	.	*SRRM2*	NS	unknown(0)	0
F9	M	Comp het	16	2817604	G	A	.	*SRRM2*	NS	possibly_damaging(0.794)	0
F9	M	Comp het	16	2817749	C	G	.	*SRRM2*	NS	probably_damaging(0.999)	0
F9	M	Comp het	2	234878910	C	T	rs17862932	*TRPM8*	NS	probably_damaging(0.996)	tolerated(0.07)
F9	M	Comp het	2	234891850	G	A	rs149328116	*TRPM8*	NS	benign(0.029)	deleterious(0)
F10	F	Comp het	19	3546264	C	T	rs34878396	*C19orf28*	NS	possibly_damaging(0.871)	tolerated(0.11)
F10	F	Comp het	19	3551120	TC	T	.	*C19orf28*	FS	0	0
F10	F	Comp het	10	85955337	C	A	.	*CDHR1*	NS	probably_damaging(0.991)	deleterious(0)
F10	F	Comp het	10	85961593	C	T	rs147420731	*CDHR1*	NS	probably_damaging(0.996)	tolerated(1)
F10	F	Comp het	12	124330648	G	A	.	*DNAH10*	NS	probably_damaging(1)	tolerated(0.57)
F10	F	Comp het	12	124413109	T	C	.	*DNAH10*	NS	probably_damaging(0.994)	deleterious(0.01)
F10	F	Comp het	1	39851427	G	A	rs145533329	*MACF1*	NS	possibly_damaging(0.798)	tolerated(0.21)
F10	F	Comp het	1	39901245	A	G	.	*MACF1*	NS	benign(0.037)	tolerated(0.15)
F11	M	Hemi	X	71521598	G	A	rs139648368	*CITED1*	NS	possibly_damaging(0.913)	tolerated(1)

F11	M	Hemi	X	3242339	T	C	rs144991234	*MXRA5*	NS	benign(0.005)	tolerated(0.3)
F11	M	Hemi	X	30322699	T	C	rs151317312	*NR0B1*	NS	benign(0.011)	deleterious(0)
F11	M	Comp het	4	57777171	C	G	rs149829250	*REST*	NS	probably_damaging(0.977)	deleterious(0)
F11	M	Comp het	4	57796913	C	T	rs144905338	*REST*	NS	benign(0)	tolerated(1)
F12	F	Comp het	20	29631562	A	G	rs75398190	*FRG1B*	NS	benign(0.002)	tolerated(0.74)
F12	F	Comp het	20	29631580	A	G	.	*FRG1B*	NS	benign(0.004)	tolerated(0.38)
F12	F	Comp het	2	179396782	C	G	rs55866005	*TTN*	NS	benign(0.003)	0
F12	F	Comp het	2	179414177	G	A	rs72648247	*TTN*	NS	probably_damaging(1)	0
F12	F	Comp het	2	179484593	C	T	rs72677229	*TTN*	NS	benign(0.014)	0
F12	F	Comp het	2	179486037	C	A	rs72677225	*TTN*	NS	benign(0.099)	0
F12	F	Comp het	2	179549707	G	A	rs72650030	*TTN*	NS	unknown(0)	0
F12	F	Comp het	2	179599473	C	G	rs72648929	*TTN*	NS	unknown(0)	0
F12	F	Comp het	6	56999585	C	A	rs140750140	*ZNF451*	NS	benign(0.137)	tolerated(0.35)
F12	F	Comp het	6	57012673	C	T	rs149876604	*ZNF451*	NS	benign(0.002)	tolerated(0.35)
F13	M	Hemi	X	110980029	G	C	rs142841538	*ALG13*	NS	benign(0.113)	tolerated(0.25)
F13	M	Hemi	X	134713929	C	G	rs77047796	*DDX26B*	NS	benign(0.006)	tolerated(0.39)
F13	M	Comp het	4	79238620	C	T	rs147869493	*FRAS1*	NS	possibly_damaging(0.643)	tolerated(0.14)
F13	M	Comp het	4	79353746	C	A	.	*FRAS1*	NS	probably_damaging(0.993)	tolerated(0.07)
F13	M	Hemi	X	135314244	G	A	.	*MAP7D3*	NS	possibly_damaging(0.918)	deleterious(0.01)
F13	M	Comp het	15	42145586	G	A	rs112040796	*SPTBN5*	NS	benign(0)	tolerated(0.36)
F13	M	Comp het	15	42154034	C	T	.	*SPTBN5*	NS	benign(0.019)	tolerated(0.7)
F13	M	Hemi	X	12904292	T	A	rs55907843	*TLR7*	NS	benign(0.196)	tolerated(0.62)
F13	M	Comp het	2	1459885	A	G	rs148683218	*TPO*	NS	benign(0.12)	tolerated(0.14)
F13	M	Comp	2	1544464	C	T	rs138289170	*TPO*	NS	possibly_damag	deleterious(0

		het								ing(0.844)	)
F14	F	Comp het	20	49508015	T	C	rs147399432	*ADNP*	NS	benign(0)	tolerated(1)
F14	F	Comp het	20	49508508	C	T	rs150900514	*ADNP*	NS	benign(0)	deleterious(0)
F14	F	Comp het	2	242144345	T	G	.	*ANO7*	NS	benign(0)	tolerated(0.81)
F14	F	Comp het	2	242162665	C	T	rs141614709	*ANO7*	NS	probably_damaging(1)	deleterious(0)
F14	F	Comp het	1	214818291	G	A	rs143725699	*CENPF*	NS	probably_damaging(0.995)	tolerated(0.6)
F14	F	Comp het	1	214819026	A	C	rs142561288	*CENPF*	NS	probably_damaging(0.959)	deleterious(0.01)
F14	F	Comp het	6	46660511	T	A	.	*TDRD6*	NS	benign(0.008)	tolerated(0.87)
F14	F	Comp het	6	46661479	G	T	rs142413497	*TDRD6*	NS	benign(0.032)	tolerated(0.25)
F15	F	Comp het	5	148586585	A	G	.	*ABLIM3*	NS	benign(0.206)	deleterious(0.01)
F15	F	Comp het	5	148627397	C	T	rs143754875	*ABLIM3*	NS	possibly_damaging(0.872)	deleterious(0.01)
F15	F	Comp het	5	82833426	A	G	rs61749614	*VCAN*	NS	benign(0.001)	0
F15	F	Comp het	5	82835589	T	C	rs146630369	*VCAN*	NS	probably_damaging(0.999)	0
F16	M	Comp het	16	1470583	C	G	rs45490596	*C16orf91*	NS	probably_damaging(0.997)	deleterious(0.01)
F16	M	Comp het	16	1476330	T	C	rs72779224	*C16orf91*	NS	unknown(0)	0
F16	M	Comp het	9	90500202	A	G	.	*C9orf79*	NS	probably_damaging(0.962)	tolerated(0.19)
F16	M	Comp het	9	90502542	T	C	.	*C9orf79*	NS	possibly_damaging(0.605)	tolerated(0.08)
F16	M	Comp het	17	20799179	C	G	.	*CCDC144NL*	NS	possibly_damaging(0.484)	deleterious(0)
F16	M	Comp het	17	20799281	G	A	rs141096337	*CCDC144NL*	NS	probably_damaging(0.996)	tolerated(0.17)
F16	M	Comp het	6	138752868	C	A	.	*NHSL1*	NS	benign(0.002)	tolerated(1)
F16	M	Comp het	6	138794490	G	A	.	*NHSL1*	NS	benign(0)	tolerated(0.5)
F16	M	Hemi	X	9863050	C	T	.	*SHROOM2*	NS	probably_damaging(0.994)	deleterious(0.03)
F17	F	Comp het	16	2369688	A	T	.	*ABCA3*	NS	probably_damaging(0.999)	deleterious(0)
F17	F	Comp	16	2374481	T	C	rs142977595	*ABCA3*	NS	possibly_damag	tolerated(0.2

		het								ing(0.731)	8)
F17	F	Comp het	13	42875878	C	T	.	*AKAP11*	NS	benign(0)	tolerated(0.5)
F17	F	Comp het	13	42876835	T	G	rs61757547	*AKAP11*	NS	benign(0.001)	tolerated(0.37)
F17	F	Comp het	1	68960131	T	C	.	*DEPDC1*	NS	probably_damaging(1)	deleterious(0.01)
F17	F	Comp het	1	68960186	T	C	.	*DEPDC1*	NS	probably_damaging(0.999)	tolerated(0.12)
F17	F	Comp het	1	26303228	G	A	rs150853838	*PAFAH2*	NS	probably_damaging(0.995)	deleterious(0)
F17	F	Comp het	1	26317303	C	T	rs148837170	*PAFAH2*	NS	probably_damaging(1)	deleterious(0)
F17	F	Comp het	7	75070377	T	A	.	*POM121C*	NS	benign(0.223)	deleterious(0.04)
F17	F	Comp het	7	75070840	C	A	.	*POM121C*	NS	possibly_damaging(0.902)	tolerated(0.17)
F18	M	Hemi	X	105855323	T	C	.	*CXorf57*	NS	possibly_damaging(0.688)	tolerated(0.17)
F18	M	Hemi	X	44703940	A	G	rs144899652	*DUSP21*	NS	benign(0.117)	tolerated(0.31)
F18	M	Hemi	X	138644189	C	T	.	*F9*	NS	benign(0.049)	deleterious(0.02)
F18	M	Hemi	X	55650995	C	T	.	*FOXR2*	NS	possibly_damaging(0.692)	tolerated(0.56)
F18	M	Hemi	X	131842557	G	C	.	*HS6ST2*	NS	benign(0.003)	deleterious(0)
F18	M	Hemi	X	119077233	C	G	.	*NKAP*	NS	unknown(0)	tolerated(0.11)
F18	M	Comp het	3	135969390	A	C	.	*PCCB*	NS	probably_damaging(0.994)	deleterious(0)
F18	M	Comp het	3	136019898	C	T	rs147538201	*PCCB*	NS	benign(0.431)	deleterious(0.01)
F18	M	Hemi	X	129546514	G	A	.	*RBMX2*	NS	benign(0)	tolerated(0.58)
F18	M	Comp het	2	179452447	T	C	rs72646855	*TTN*	NS	benign(0.014)	0
F18	M	Comp het	2	179611552	C	T	rs62179016	*TTN*	NS	possibly_damaging(0.888)	tolerated(0.33)
F18	M	Comp het	16	72831357	C	CTTGTTG	rs34918837	*ZFHX3*	NS	0	0
F18	M	Comp het	16	72831629	G	A	rs117283459	*ZFHX3*	NS	unknown(0)	0
F18	M	Comp het	16	72832550	A	C	rs145446485	*ZFHX3*	NS	unknown(0)	0
F19	M	Hom	16	84229207	C	T	rs138702097	*ADAD2*	NS	probably_dama	tolerated(0.1

										ging(0.996)	2)
F19	M	Comp het	14	105415079	G	T	.	*AHNAK2*	NS	probably_damaging(0.996)	tolerated(0.2)
F19	M	Comp het	14	105416541	C	G	.	*AHNAK2*	NS	possibly_damaging(0.616)	tolerated(0.47)
F19	M	Comp het	20	61326565	C	T	.	*C20orf90*	NS	unknown(0)	0
F19	M	Comp het	20	61331818	C	G	rs147473138	*C20orf90*	NS	unknown(0)	0
F19	M	Comp het	12	7521535	A	G	rs145256685	*CD163L1*	NS	possibly_damaging(0.681)	0
F19	M	Comp het	12	7527284	C	T	rs36206713	*CD163L1*	NS	possibly_damaging(0.859)	deleterious(0)
F19	M	Hemi	X	107431191	T	A	.	*COL4A6*	NS	benign(0.049)	tolerated(0.73)
F19	M	Comp het	3	52409413	C	G	rs144580984	*DNAH1*	NS	probably_damaging(0.988)	tolerated(0.16)
F19	M	Comp het	3	52426643	G	A	.	*DNAH1*	NS	possibly_damaging(0.734)	deleterious(0.01)
F19	M	Comp het	5	13759007	G	A	rs149956015	*DNAH5*	NS	benign(0.002)	tolerated(0.27)
F19	M	Comp het	5	13883075	C	T	rs146828513	*DNAH5*	NS	benign(0.275)	tolerated(0.09)
F19	M	Comp het	2	84880481	C	G	.	*DNAH6*	NS	probably_damaging(0.981)	deleterious(0.03)
F19	M	Comp het	2	84924743	C	G	.	*DNAH6*	NS	probably_damaging(1)	deleterious(0.01)
F19	M	Comp het	5	132534965	G	A	rs143403129	*FSTL4*	NS	possibly_damaging(0.538)	tolerated(0.22)
F19	M	Comp het	5	132939589	C	T	.	*FSTL4*	NS	benign(0.147)	deleterious(0)
F19	M	Hemi	X	2793951	T	C	rs148872483	*GYG2*	NS	benign(0)	tolerated(0.38)
F19	M	Hom	21	47831802	C	T	.	*PCNT*	NS	probably_damaging(1)	deleterious(0)
F19	M	Comp het	16	71712805	C	A	.	*PHLPP2*	NS	probably_damaging(0.994)	tolerated(0.16)
F19	M	Comp het	16	71724598	T	C	.	*PHLPP2*	NS	benign(0)	tolerated(1)
F19	M	Hemi	X	152225801	G	A	.	*PNMA3*	NS	benign(0)	tolerated(1)
F19	M	Hemi	X	84362764	G	A	rs150924046	*SATL1*	NS	benign(0.001)	tolerated(0.13)
F19	M	Hemi	X	9863131	G	A	.	*SHROOM2*	NS	benign(0.327)	tolerated(0.68)
F20	M	Comp het	8	61769198	C	G	.	*CHD7*	NS	probably_damaging(0.959)	deleterious(0.01)

F20	M	Comp het	8	61778448	C	T	.	*CHD7*	NS	probably_damaging(0.978)	tolerated(0.09)
F20	M	Comp het	6	131247845	T	A	.	*EPB41L2*	NS	benign(0)	tolerated(0.07)
F20	M	Comp het	6	131277390	G	A	rs144686133	*EPB41L2*	NS	probably_damaging(1)	tolerated(0.06)
F20	M	Hemi	X	152860096	C	T	rs150562029	*FAM58A*	NS	benign(0.008)	deleterious(0.02)
F20	M	Comp het	5	90002053	A	G	rs41308297	*GPR98*	NS	benign(0)	tolerated(1)
F20	M	Comp het	5	90059270	C	A	.	*GPR98*	NS	probably_damaging(1)	tolerated(0.15)
F20	M	Hemi	X	154290176	C	G	rs146554247	*MTCP1NB*	NS	probably_damaging(1)	deleterious(0)
F20	M	Hemi	X	153697736	C	T	rs140345657	*PLXNA3*	NS	probably_damaging(0.99)	deleterious(0.02)
F20	M	Hemi	X	153716622	T	C	.	*SLC10A3*	NS	benign(0)	tolerated(0.29)
F20	M	Comp het	1	12337460	T	A	.	*VPS13D*	NS	probably_damaging(0.98)	tolerated(0.07)
F20	M	Comp het	1	12337667	C	T	rs12407578	*VPS13D*	NS	probably_damaging(0.973)	deleterious(0.04)
F20	M	Comp het	1	12378274	C	T	rs150598243	*VPS13D*	NS	probably_damaging(0.982)	tolerated(0.14)
F21	M	Hemi	X	100911707	G	A	rs143836164	*ARMCX2*	NS	probably_damaging(0.999)	deleterious(0.02)
F21	M	Comp het	16	1265315	G	A	rs148651456	*CACNA1H*	NS	probably_damaging(1)	tolerated(0.14)
F21	M	Comp het	16	1270350	G	A	rs59487037	*CACNA1H*	NS	benign(0)	tolerated(0.24)
F21	M	Hemi	X	65824281	G	A	rs12837393	*EDA2R*	NS	probably_damaging(0.999)	deleterious(0.01)
F21	M	Hemi	X	135593768	G	A	.	*HTATSF1*	NS	benign(0.158)	deleterious(0)
F21	M	Hom	14	104641986	C	G	.	*KIF26A*	NS	probably_damaging(0.991)	deleterious(0)
F21	M	Hemi	X	135303057	T	C	.	*MAP7D3*	NS	benign(0.047)	tolerated(0.4)
F21	M	Hemi	X	63490871	TC	T	.	*MTMR8*	FS	0	0
F21	M	Hemi	X	3239828	T	C	rs41297257	*MXRA5*	NS	benign(0)	deleterious(0.03)
F21	M	Comp het	6	51656129	C	G	rs147222255	*PKHD1*	NS	probably_damaging(0.99)	tolerated(0.55)
F21	M	Comp het	6	51768399	A	T	.	*PKHD1*	NS	possibly_damaging(0.775)	deleterious(0.03)
F22	M	Comp het	8	91049129	C	G	.	*DECR1*	NS	probably_damaging(1)	deleterious(0.03)

F22	M	Comp het	8	91057198	A	G	rs148549954	*DECR1*	NS	benign(0.32)	tolerated(0.09)
F22	M	Comp het	15	45411495	C	A	rs148849457	*DUOXA1*	NS	benign(0.137)	tolerated(0.11)
F22	M	Comp het	15	45412435	G	A	rs149960164	*DUOXA1*	NS	probably_damaging(1)	deleterious(0)
F22	M	Hemi	X	135314244	G	A	.	*MAP7D3*	NS	possibly_damaging(0.918)	deleterious(0.01)
F22	M	Comp het	2	152346522	G	A	rs78592085	*NEB*	NS	probably_damaging(1)	0
F22	M	Comp het	2	152384078	C	T	.	*NEB*	NS	probably_damaging(0.997)	tolerated(0.1)
F22	M	Hom	5	140553876	T	C	rs116101007	*PCDHB7*	NS	benign(0)	tolerated(1)
F22	M	Comp het	15	62212467	C	T	rs143926369	*VPS13C*	NS	benign(0.009)	tolerated(0.55)
F22	M	Comp het	15	62212770	T	C	rs139993005	*VPS13C*	NS	benign(0)	tolerated(0.58)
F23	M	Comp het	1	170952626	T	C	.	*C1orf129*	NS	benign(0.009)	tolerated(0.7)
F23	M	Comp het	1	170961328	C	T	rs146036672	*C1orf129*	NS	probably_damaging(0.998)	deleterious(0.02)
F23	M	Hom	5	74018232	A	G	rs143306569	*GFM2*	NS	benign(0)	tolerated(0.64)
F23	M	Hemi	X	19398315	C	T	rs56381411	*MAP3K15*	NS	probably_damaging(0.994)	deleterious(0.01)
F23	M	Hemi	X	135313855	T	C	.	*MAP7D3*	NS	probably_damaging(0.99)	deleterious(0.01)
F23	M	Comp het	11	70332311	C	T	.	*SHANK2*	NS	possibly_damaging(0.72)	tolerated(0.19)
F23	M	Comp het	11	70336479	C	T	rs117843717	*SHANK2*	NS	probably_damaging(0.993)	tolerated(0.08)
F23	M	Comp het	2	179404498	G	C	rs72648273	*TTN*	NS	probably_damaging(0.992)	0
F23	M	Comp het	2	179424272	C	A	.	*TTN*	NS	possibly_damaging(0.862)	0
F23	M	Comp het	2	179454530	C	T	.	*TTN*	NS	benign(0.287)	0
F23	M	Comp het	2	179610967	C	T	rs72648913	*TTN*	NS	benign(0.334)	deleterious(0)
F25	M	Hemi	X	39932564	C	T	rs144722432	*BCOR*	NS	possibly_damaging(0.931)	deleterious(0)
F25	M	Comp het	1	22150156	G	T	rs3736359	*HSPG2*	NS	probably_damaging(0.998)	deleterious(0)
F25	M	Comp het	1	22206977	C	T	rs143669458	*HSPG2*	NS	probably_damaging(0.997)	deleterious(0.04)
F25	M	Comp	1	156497776	C	CA	.	*IQGAP3*	FS	0	0

		het									
F25	M	Comp het	1	156504308	G	A	rs147048069	*IQGAP3*	NS	benign(0.003)	tolerated(0.06)
F25	M	Hemi	X	102755132	TC	T	.	*RAB40A*	FS	0	0
F25	M	Hemi	X	132160102	G	A	.	*USP26*	NS	benign(0)	tolerated(0.24)
F26	M	Comp het	1	145515394	A	G	rs61746197	*GNRHR2*	NS	benign(0)	deleterious(0.01)
F26	M	Comp het	1	145515696	A	T	rs72701872	*GNRHR2*	NS	unknown(0)	0
F26	M	Hemi	X	135593322	A	G	rs149350146	*HTATSF1*	NS	benign(0.074)	tolerated(0.44)
F26	M	Hemi	X	149931185	G	A	rs77346702	*MTMR1*	NS	benign(0.243)	tolerated(0.43)
F26	M	Hemi	X	15474123	G	T	rs147114611	*PIR*	NS	benign(0)	tolerated(0.66)
F28	F	Comp het	20	52773992	C	T	rs112596218	*CYP24A1*	NS	benign(0)	tolerated(0.92)
F28	F	Comp het	20	52788189	C	T	rs35051736	*CYP24A1*	NS	possibly_damaging(0.846)	tolerated(0.2)
F28	F	Comp het	4	123179882	T	G	.	*KIAA1109*	NS	probably_damaging(0.993)	tolerated(0.1)
F28	F	Comp het	4	123207867	T	G	rs79067453	*KIAA1109*	NS	benign(0)	tolerated(0.11)
F28	F	Comp het	16	84514205	G	A	rs113834725	*KIAA1609*	NS	probably_damaging(1)	deleterious(0)
F28	F	Comp het	16	84516214	G	A	rs140439420	*KIAA1609*	NS	probably_damaging(1)	deleterious(0)
F28	F	Comp het	17	70845790	G	A	rs144832523	*SLC39A11*	NS	probably_damaging(0.999)	deleterious(0.01)
F28	F	Comp het	17	70944008	C	T	rs34970573	*SLC39A11*	NS	benign(0)	tolerated(0.51)
F29	F	Comp het	7	48312484	A	G	.	*ABCA13*	NS	benign(0.002)	0
F29	F	Comp het	7	48313854	G	A	.	*ABCA13*	NS	possibly_damaging(0.798)	0
F29	F	Comp het	3	182923984	G	A	rs61750384	*MCF2L2*	NS	probably_damaging(0.997)	deleterious(0)
F29	F	Comp het	3	183097166	G	A	.	*MCF2L2*	NS	probably_damaging(1)	deleterious(0.01)
F29	F	Comp het	19	54301638	G	C	rs146671776	*NLRP12*	NS	probably_damaging(0.981)	tolerated(0.37)
F29	F	Comp het	19	54314254	C	T	.	*NLRP12*	NS	probably_damaging(1)	deleterious(0)
F29	F	Comp het	7	75052435	C	T	.	*POM121C*	NS	possibly_damaging(0.873)	tolerated(0.11)

F29	F	Comp het	7	75070334	C	T	.	*POM121C*	NS	benign(0.113)	deleterious(0)
F29	F	Hom	2	179396782	C	G	rs55866005	*TTN*	NS	benign(0.003)	0
F29	F	Comp het	2	179454969	G	A	.	*TTN*	NS	possibly_damaging(0.737)	0
F29	F	Hom	2	179486037	C	A	rs72677225	*TTN*	NS	benign(0.099)	0
F29	F	Comp het	2	179582913	C	T	rs72648981	*TTN*	NS	unknown(0)	0
F29	F	Comp het	20	57766294	C	G	.	*ZNF831*	NS	probably_damaging(0.998)	deleterious(0)
F29	F	Comp het	20	57769291	C	T	.	*ZNF831*	NS	benign(0.002)	deleterious(0.02)
F31	F	Comp het	15	80452844	G	A	rs146263676	*FAH*	NS	possibly_damaging(0.815)	deleterious(0)
F31	F	Comp het	15	80464527	C	A	.	*FAH*	NS	benign(0.082)	tolerated(0.06)
F33	F	Comp het	1	981151	T	C	.	*AGRN*	NS	probably_damaging(0.999)	deleterious(0)
F33	F	Comp het	1	985378	G	A	rs147259096	*AGRN*	NS	benign(0.224)	tolerated(1)
F33	F	Comp het	19	33183575	T	A	.	*NUDT19*	NS	possibly_damaging(0.585)	tolerated(0.19)
F33	F	Comp het	19	33200127	T	C	.	*NUDT19*	NS	probably_damaging(0.993)	tolerated(0.17)

Table S3: High-quality, rare, coding, inherited recessive and X-linked SNPs and indels. FS= frameshift coding NS = non-synonymous CQ = consequence of mutation; GDR = gender of fetus; GT = genotype

ID	CHR	Start position	End position	Size (kb)	Number probes	CoNVex score	CNV type	Inheritance model	GN	Category
F14	X	13770686	13791294	20.6	15	26	DEL	*de novo*	*GPM6B; OFD1*	Highly likely to be causal
F19	X	48155306	48270940	115.6	32	68	DUP	Inherited X linked	*SSX3; SSX4; SSX4B*	Unknown
F3	X	103267111	103301913	34.8	5	17	DUP	Inherited X linked	*H2BFM; H2BFWT*	Unknown

Table S4: Details of CNVs called by CoNVex in fetuses with structural abnormalities. None of the genes in these CNVs have additional mutations likely to cause disease. None of these CNVs have any overlap with common CNVs. The deletion in F14 was confirmed by aCGH. GN = protein coding genes.

References

1 Hillman, S.C., McMullan, D.J., Hall, G., Togneri, F.S., James, N., Maher, E.J., Meller, C.H., Williams, D., Wapner, R.J., Maher, E.R. *et al.* (2013) Use of prenatal chromosomal microarray: prospective cohort study and systematic review and meta-analysis. *Ultrasound Obstet. Gynecol.*, **41**, 610-620.

2 DePristo, M.A., Banks, E., Poplin, R., Garimella, K.V., Maguire, J.R., Hartl, C., Philippakis, A.A., del Angel, G., Rivas, M.A., Hanna, M. *et al.* (2011) A framework for variation discovery and genotyping using next-generation DNA sequencing data. *Nat. Genet.*, **43**, 491-498.

3 Li, H., Handsaker, B., Wysoker, A., Fennell, T., Ruan, J., Homer, N., Marth, G., Abecasis, G., Durbin, R. and Genome Project Data Processing Subgroup (2009) The Sequence Alignment/Map format and SAMtools. *Bioinformatics*, **25**, 2078-2079.

4 Albers, C.A., Lunter, G., MacArthur, D.G., McVean, G., Ouwehand, W.H. and Durbin, R. (2011) Dindel: accurate indel calls from short-read data. *Genome Res.*, **21**, 961-973.

5 Flicek, P., Ahmed, I., Amode, M.R., Barrell, D., Beal, K., Brent, S., Carvalho-Silva, D., Clapham, P., Coates, G., Fairley, S. *et al.* (2013) Ensembl 2013. *Nucleic Acids Res.*, **41**, D48-55.

6 Genomes Project Consortium, Abecasis, G.R., Auton, A., Brooks, L.D., DePristo, M.A., Durbin, R.M., Handsaker, R.E., Kang, H.M., Marth, G.T. and McVean, G.A. (2012) An integrated map of genetic variation from 1,092 human genomes. *Nature*, **491**, 56-65.

7 Adzhubei, I.A., Schmidt, S., Peshkin, L., Ramensky, V.E., Gerasimova, A., Bork, P., Kondrashov, A.S. and Sunyaev, S.R. (2010) A method and server for predicting damaging missense mutations. *Nat. Methods*, **7**, 248-249.

8 Kumar, P., Henikoff, S. and Ng, P.C. (2009) Predicting the effects of coding non-synonymous variants on protein function using the SIFT algorithm. *Nat. Protoc.*, **4**, 1073-1081.

9 Conrad, D.F., Keebler, J.E., DePristo, M.A., Lindsay, S.J., Zhang, Y., Casals, F., Idaghdour, Y., Hartl, C.L., Torroja, C., Garimella, K.V. *et al.* (2011) Variation in genome-wide mutation rates within and between human families. *Nat. Genet.*, **43**, 712-714.

10 Ramu, A., Noordam, M.J., Schwartz, R.S., Wuster, A., Hurles, M.E., Cartwright, R.A. and Conrad, D.F. (2013) DeNovoGear: de novo indel and point mutation discovery and phasing. *Nat. Methods*, **10**, 985-987.

11 Thorvaldsdottir, H., Robinson, J.T. and Mesirov, J.P. (2013) Integrative Genomics Viewer (IGV): high-performance genomics data visualization and exploration. *Brief Bioinform*, **14**, 178-192.

12 Kryukov, G.V., Pennacchio, L.A. and Sunyaev, S.R. (2007) Most rare missense alleles are deleterious in humans: implications for complex disease and association studies. *Am. J. Hum. Genet.*, **80**, 727-739.

13 Rauch, A., Wieczorek, D., Graf, E., Wieland, T., Endele, S., Schwarzmayr, T., Albrecht, B., Bartholdi, D., Beygo, J., Di Donato, N. *et al.* (2012) Range of genetic mutations associated with severe non-syndromic sporadic intellectual disability: an exome sequencing study. *Lancet*, **380**, 1674-1682.

14 Danecek, P., Auton, A., Abecasis, G., Albers, C.A., Banks, E., DePristo, M.A., Handsaker, R.E., Lunter, G., Marth, G.T., Sherry, S.T. *et al.* (2011) The variant call format and VCFtools. *Bioinformatics*, **27**, 2156-2158.

15 Amarasinghe, K.C., Li, J. and Halgamuge, S.K. (2013) CoNVEX: copy number variation estimation in exome sequencing data using HMM. *BMC Bioinformatics*, **14 Suppl 2**, S2.
